# Collagen reorganization in cartilage under strain probed by polarization sensitive second harmonic generation microscopy

**DOI:** 10.1098/rsif.2018.0611

**Published:** 2019-01-16

**Authors:** Jessica C. Mansfield, Vipul Mandalia, Andrew Toms, C. Peter Winlove, Sophie Brasselet

**Affiliations:** 1 Physics, College of Engineering Mathematics and Physical Sciences, University of Exeter, Stocker Road, Exeter EX4 4QL, UK; 2 Princess Elizabeth Orthopaedic Centre, Royal Devon and Exeter Hospital, Barrack Road, Exeter EX2 5DW, UK; 3 Institut Fresnel, CNRS, Aix Marseille Univ, Centrale Marseille, 13013 Marseille, France

**Keywords:** cartilage, collagen, second harmonic generation, polarization, micromechanics

## Abstract

Type II collagen fibril diameters in cartilage are beneath the diffraction limit of optical microscopy, which makes the assessment of collagen organization very challenging. In this work we use polarization sensitive second harmonic generation (P-SHG) imaging to map collagen organization in articular cartilage, addressing in particular its behaviour under strain and changes which occur in osteoarthritis. P-SHG yields two parameters, molecular order and orientation, which provide measures of the degree of organization both at the molecular scale (below the diffraction limit) and above a few hundred nanometres (at the image pixel size). P-SHG clearly demonstrates the zonal collagen architecture and reveals differences in the structure of the fibrils around chondrocytes. P-SHG also reveals sub-micron scale fibril re-organization in cartilage strips exposed to tensile loading, with an increase in local organization in the superficial zone which weakly correlates with tensile modulus. Finally, P-SHG is used to investigate osteoarthritic cartilage from total knee replacement surgery, and reveals widespread heterogeneity across samples both microscale fibril orientations and their sub-micron organization. By addressing collagen fibril structure on scales intermediate between conventional light and electron microscopy, this study provides new insights into collagen micromechanics and mechanisms of degradation.

## 1. Background

**Figure 1.**
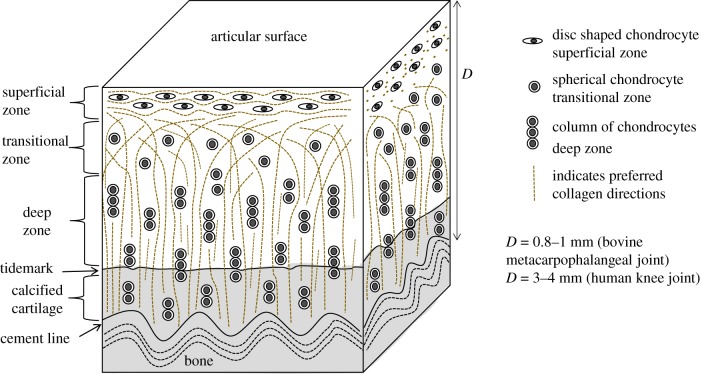
Collagen fibre arrangements in cartilage showing the zonal structure of articular cartilage. (Online version in colour.)

Articular cartilage covers the ends of the long bones in synovial joints where it acts as a low friction bearing and shock absorber which is essential to the smooth articulation of the joint [1]. Cartilage has a low cellular content, typically around 1–10%, and the extracellular matrix which fulfils the tissue's mechanical role is composed of approximately 20% type II collagen, 8% proteoglycans and 70% water. The collagen fibril arrangement changes with depth into the tissue as schematically depicted in figure 1, and can be divided into three zones. In the superficial zone the fibrils are arranged predominantly parallel to the articular surface, in the deep zone they are arranged perpendicular to the articular surface and the bone cartilage interface and between these two zones there is a transitional zone with less clearly defined collagen fibril orientation. The type II collagen fibrils are typically 30–200 nm in diameter [2] and therefore just below the diffraction limit for conventional light microscopy.

The mechanical properties of cartilage are highly dependent on the organization of the collagen fibrils. Under tensile load the cartilage is much stiffer in the superficial zone compared to the deep zone [3,4], additionally it is much stiffer in the direction parallel to the split lines [4,5] (an indication of the preferred collagen fibril direction in the superficial zone). The tensile modulus also varies between high and low weight bearing regions [6] and decreases with osteoarthritic degeneration and age [7].

The mechanical properties of cartilage on a much smaller scale are central to the processes of mechanotransduction to the embedded chondrocytes that maintain matrix homeostasis and are strongly implicated in the development of osteoarthritis (OA). However, recent work has revealed that on the microscale the mechanical response of articular cartilage to tensile loads is more heterogeneous than previously appreciated and the relationship to collagen architecture is extremely complex [8,9]. On a still smaller scale it has been shown that the intrafibrillar organization of cartilage collagen varies between zones and, importantly, changes under mechanical loads. Electron microscopy has provided vital information on the sub-micron organization of cartilage collagen and has revealed structural changes associated with the development of OA [10–13]. It has also shown differences in structure between mechanically loaded and unloaded cartilage [11], but it cannot be used to follow the dynamics of mechanical loading as required to understand the mechanical implications of degenerative changes. The aim of the present work was to bridge the gap between classical light microscopy and electron microscopy, using an extension of multiphoton microscopy that exploits the polarization-sensitivity of the signal to provide resolution beyond the diffraction limit and enables us to explore cartilage collagen morphology and organization in intact tissues on a sub-micron scale.

The birefringence of collagen has long been recognized and was the basis of Bennighoff's classical studies on cartilage structure. However, although polarized light microscopy reveals fibril organization with a sub-micron lateral resolution it relies on optical measurements through sections of tissues in which the information is averaged over the whole thickness, which makes it insensitive to volumetric organization and unsuited to quantitative analysis of responses to mechanical loads [14,15]. Polarized optical coherence tomography (OCT) allows the analysis of specific image planes but it suffers from a poor axial resolution of a few tens of microns [16,17].

In contrast, second harmonic generation (SHG) which exploits the non-centrosymmetric, tightly packed arrangement of amino acids in collagen to provide label-free imaging has sub-micrometre 3D sectioning capability and in-depth penetration in tissues up to a few hundreds of micrometres using infrared excitation and has become a gold standard technique for imaging collagen in tissues [18,19]. SHG imaging can also reveal collagen orientation, by the analysis of 3D volumetric images to extract directional information using gradient, FFT or variance analysis. This approach however requires high contrast images over small length scales and depends strongly on the choice of the analysis parameters [20–22], which makes it challenging to apply to type II collagen tissues such as cartilage [23]. An alternative solution to image fine type II collagen fibrils has been proposed to be analysis of the ratio between forward scattered and backward emitted SHG which is sensitive to collagen fibrils size and density, however the measurements are only qualitatively related to organization and therefore also unsuited to our present purpose [23,24].

In this work, we exploit the polarization sensitivity of SHG at scales below the optical diffraction limit (typically 350 nm lateral and 1 µm axial) to assess collagen organization in cartilage. The intensity of SHG from a collagen fibre is dependent on the polarization of the laser source with respect to the axis of the fibre [25–27]. Measuring SHG intensity as a function of the incident laser polarization angle yields a modulated response where the phase determines the principal direction of collagen fibrils averaged within the focal volume, and the amplitude is related to the degree of alignment of the collagen fibrils within this focal volume. This modulation permits quantification of collagen angular disorder at the molecular scale [28–34], which cannot be determined by pure morphological observations, which only report orientation at a scale above the diffraction limit size. Polarization sensitive SHG (P-SHG) has previously been used to quantify collagen order in tendons [27,35] and other connective tissues [32,36] has revealed variations of collagen order during ageing [37], and in pathologies including cancer [18,38,39]. Very few studies, however, address changes in P-SHG from collagen under strain [40], or in cartilage [41,42]. Importantly, P-SHG provides information on collagen fibril organization on a scale intermediate between those of conventional light and electron microscopy, which is particularly relevant in cartilage where collagen II forms fibrils of small scales that result in poorly defined structures in an optical image. By adding P-SHG as an additional modality to SHG imaging, we show that it is possible to decipher molecular organization in collagen networks even though the structures are not well defined from the pure SHG image.

In this paper, we use P- SHG to investigate differences in submicron scale collagen fibril organization between the deep and superficial zones of articular cartilage. We moreover report variations of collagen organization in the matrix surrounding the chondrocytes both in healthy bovine cartilage and in human knee cartilage removed during arthroplasty. Finally, we show that tensile strain applied to the cartilage gives rise to changes in the submicron organization of the fibrils, particularly in the superficial zone.

## 2. Material and methods

### 2.1. Sample preparation

Bovine cartilage was taken from the metacarpophalangeal joint of nine bovine forelimbs collected from the local abattoir with an age range of 24–30 months. The cartilage was harvested immediately and stored at −20°C prior to use. Human cartilage samples were collected from patients undergoing total knee replacement surgery. Potential participants were initially identified during routine clinical practice, and subsequently recruited to the Royal Devon and Exeter Tissue Bank (RDETB). The RDETB is an ethically approved tissue bank (REC no.: 16//SC/0162) set up to collect ‘spare’ tissue at the time of routine clinical procedures. The total knee replacement tissue collection specific to this project was subject to review and approval by the RDETB Scientific Steering Committee as part of the overarching RDETB ethical approval. Recruitment and sample/data collection was carried out by the RDETB team. Anonymized samples and associated data are then provided to the research team via a standard material transfer agreement. The samples were assessed and graded for OA on the Outerbridge scale [43] by the surgeon and stored at −80°C prior to analysis. For both bovine and human samples enface slices of deep and superficial zone cartilage were removed from the bone using a scalpel blade and full depth sections were prepared using a purpose made cutter.

### 2.2. Multiphoton microscopy

SHG imaging was carried out using a modified confocal microscope (Olympus Fluoview 300 BX51). The SHG was excited with the 810 nm output of a 100 fs pulsed Ti:sapphire laser (Coherent MIRA 900). The light was focused on the sample using a long working distance 1.05 NA water immersion lens (Olympus XLPLN25XWMP), giving an expected minimum focal spot diameter of 350 nm. The laser power in the focal plane was 30 mW. SHG and TPF were collected from the samples in the backscattered direction. The signal was separated from the laser fundamental by a long pass dichroic filter (670dcxr Chroma technologies) and a blue green colour glass filter (CG-BG-39 CVI laser) and the SHG and TPF were directed onto two separate photomultiplier tubes (PMTs) (R3896 Hamamatsu Japan) by a long pass dichroic filter (Semrock Di02-R405). Additional filters, (Semrock FF01-405/10) and (Semrock FF01-520/70) respectively, were placed in front of the SHG and TPF PMTs.

To perform polarization dependent SHG (P-SHG) imaging, a half wave plate (WPH05M-488, Thorlabs, Newton, NJ) was placed in a motorized rotating mount (PR50CC, Newport, Irvine, CA) before the entrance to the scan unit. The polarization of the incident laser beam was rotated from 0° to 162° in 18° steps and an SHG image was taken at each rotation angle. The image sizes were 512 × 512 pixels. When establishing the measurements the polarization of the laser beam was carefully checked in the position of the microscope objective, here we found that no noticeable ellipticity or diattenuation had been introduced by the microscope optics at any incident polarization angle.

### 2.3. Polarization sensitivity analysis

The SHG intensity depends on the fourth power of the incident field *E*(*α*), whose coordinates are (cos *α*, sin *α*) in the (*X*,*Y*) sample plane, where *α* is the angle of the linear polarization with respect to the horizontal axis *X*. As a first approximation, the longitudinal polarization contribution of the focused beam is considered negligible since the fibrils lie principally in the sample plane and in-plane nonlinear coupling is predominant [30]. The nonlinear coupling of SHG leads to a fourth order dependence of the intensity *I*(*α*) on *α*, written as [30]:

2.1 $$I(\alpha )=a_{0}+a_{2}\cos2\alpha +b_{2}\sin2\alpha +a_{4}\cos4\alpha +b_{4}\sin4\alpha .$$

*I*(*α*) is analysed for each pixel of the SHG image by calculating the coefficients (*a*_0_, *a*_2_, *b*_2_, *a*_4_, *b*_4_) by projection on their corresponding circular functions [30]. These coefficients can be grouped into second and fourth order responses by rewriting the *I*(*α*) dependence into:

2.2 $$I(\alpha )=a_{0}+I_{2}\cos(2(\alpha -\phi_{2}))+I_{4}\cos(4(\alpha -\phi_{4}))$$

with:

2.3a $$I_{2}=\;\frac{\sqrt{a_{2}^{2}+b_{2}^{2}}}{a_{0}}\;,\;$$

2.3b $$I_{4}=\;\frac{\sqrt{a_{4}^{2}+b_{4}^{2}}}{a_{0}}\;,$$

2.3c $$\varphi_{2}=0.5\;\tan^{-1}\left(\frac{b_{2}}{a_{2}}\right)\;\;$$

and

2.3d $$\;\;\varphi_{4}=0.25\;\tan^{-1}\left(\frac{b_{4}}{a_{4}}\right).$$

The second order parameters (*I*_2_, *φ*_2_) represent the magnitude and orientation of the anisotropic contribution to the polarization response *I*(*α*), which quantify the depth of modulation of the polarization SHG response (*I*_2_) and its phase (*φ*_2_). (*I*_4_, *φ*_4_) are the magnitude and orientation signatures of its more complex fourth order dependence. These parameters can be related to the angular distribution of the nonlinear induced dipoles in the sample plane [30,34,37], which is directly related to the orientational organization of collagen fibres. Alternatively, they can be related to the partial determination of the nonlinear tensor at the origin of the SHG signal [26,27,31–33,40]. However since the exact model for the collagen fibril distribution in the cartilage tissues is not known, we focus in this study on the changes and variations in *I*_2_ and *φ*_2_. These parameters are generic indicators of the preferred collagen fibril alignment (*φ*_2_) and the degree of order of the individual collagen fibrils within the focal spot (*I*_2_), without the need to invoke a specific model for their angular distribution. Higher values of *I*_2_ indicate, in particular, a tighter alignment of the nonlinear induced dipole sources of SHG, presumed to reflect the distribution of peptide bonds along the protein backbones of the collagen fibrils [44] (see electronic supplementary material 1). Imaging the (*I*_2_, *φ*_2_) parameters therefore permits mapping not only the local molecular organization within collagen fibrils assemblies, but also their microscopic scale orientation. Note that the *I*_4_ parameter gives more refined information on the angular distribution experienced by the nonlinear induced dipoles in the focal spot: in particular it discriminates between distribution shapes such as Gaussian, cone or cone surface [30] (see electronic supplementary material 1). The *φ*_4_ angle finally identifies possible deviations from a cylindrical symmetric response, which can occur when the incident polarization is affected by the birefringence of the medium (see electronic supplementary material 2).

**Figure 2.**
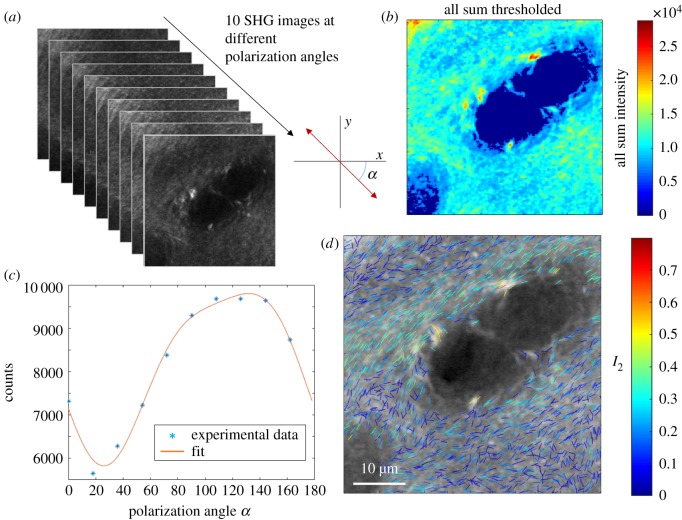
Polarization sensitive SHG data acquisition and analysis. (*a*) Series of SHG images are taken at angles from 0–162° at 18° intervals. (*b*) The intensity sum over all polarization angles is calculated, and the data are thresholded to exclude areas with low signal. For each pixel the equation (2.1) is fitted to the polarization data as a function of angle *α* relative to the horizontal axis of the sample plane. An example plot is shown in (*c*). From these data the parameters *I*_2_, *φ*_2_, *I*_4_ and *φ*_4_ can be calculated. The organization of collagen fibres is displayed as shown in (*d*) where the direction of the lines represents collagen fibre alignment (*φ*_2_) and the line colour represents the degree of organization *I*_2_ (higher *I*_2_ representing more ordered collagen fibres). To aid visualization only every 13th pixels is represented by a line in the direction of *φ*_2_. (Online version in colour.)

The spatial distributions of the parameters (*I*_2_, *φ*_2_) that represent collagen fibril organization are presented below as an overlay as shown in figure 2, where the total SHG intensity (sum over all polarization angles) is shown as a grey-scale background. The direction of the lines in the overlay represents the angle *φ*_2_ and the colour of the lines represents the value of *I*_2_ as shown in the colour bar scale (all lines are of equal length). *I*_2_ and *φ*_2_ are calculated for each pixel, however in the overlay only the *n*th lines are plotted to allow easier visualization and separation between individual lines.

#### 2.3.1. Sensitivity to birefringence

As mentioned above, the presence of birefringence in the sample can lead to distortions of the incident polarization and therefore to a bias of the parameters determined from a P-SHG polarization modulation measurement. In collagen-rich tissues, an anisotropic organization imposes a preferential optical axis along which the refractive index is larger, yielding a birefringence effect that has been used as a read-out for collagen organization. When propagating through thin depths this birefringence is, however, not dominant and the measured SHG polarization response is not sensitive to index change, but rather to the fine details of collagen orientation. At large depth however, birefringence can not only distort the incident polarization but also lead to large errors in the determined orientation parameters, in particular when the birefringence axis is rotating throughout the depth of propagation. To avoid such birefringence effects the measurements were restricted to the top 15 μm of the cartilage depth, where birefringence was found to have only a minimal effect on the polarization and SHG polarization sensitivity (see electronic supplementary material 2).

#### 2.3.2. Sensitivity to out-of-plane orientation

Working under high numerical aperture (NA) excitation and collection is known to introduce extra polarization coupling contributions from out of plane components of the dipole orientations [36,45]. In a typical regime of cylindrical symmetry with tensorial components similar to those measured in collagen tissues, this coupling is, however, negligible until off-plane tilt orientations of the collagen fibrils around 50° (see electronic supplementary material 3). Quantitative analysis of collagen organization using P-SHG can thus be done only in situations where the fibres are known to lie in the sample plane within this angular range.

### 2.4. Tensile loading

Cartilage strips were exposed to tensile loading in a rig designed to fit onto the stage of the multiphoton microscope, which is described in [9]. Strips of cartilage 200–500 µm thick (10 mm × 1 mm) were cut parallel to the articular surface, and attached with cyanoacrylate adhesive (Loctite) to two metal paddles. Strain (Δ*l*/*l*_0_ where *l*_0_ is the initial free length of cartilage between the paddles) was applied in 4% steps using a pair or micrometre controlled stages (Thorlabs). The load was continuously monitored via a 5 N force transducer (Model 31, RDP Electronics) from these measurements the stress (*F*/*A*) was calculated where F is the force measured after the sample has reached equilibrium and A is the cross-sectional area of the unstrained strip. After force equilibration at each strain a set of polarization sensitive SHG measurements were taken.

## 3. Results

### 3.1. Collagen architecture

**Figure 3.**
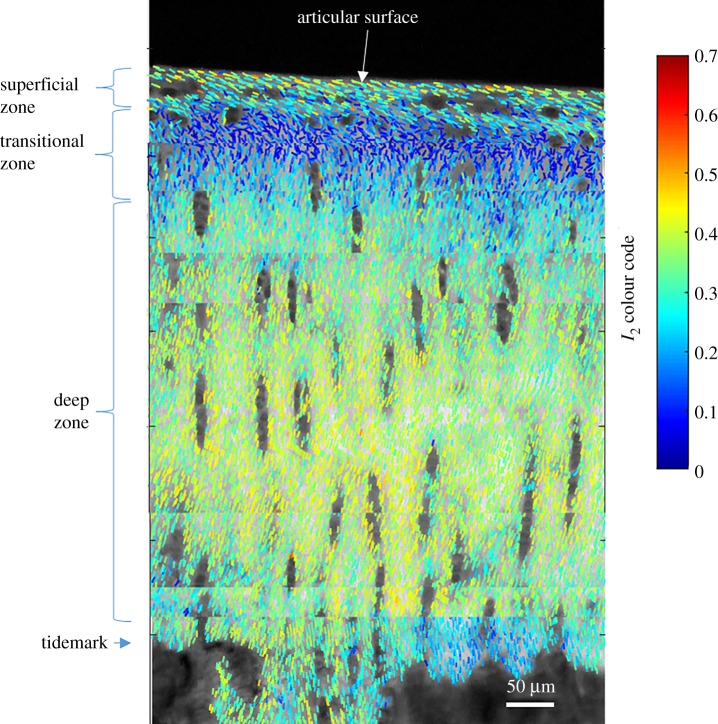
(*I*_2_, *φ*_2_) map showing collagen fibre organization in a sagittal section of bovine articular cartilage, showing the arcade structure of collagen fibres as originally described by Benninghoff [46]. (Online version in colour.)

P-SHG analysis was first performed on full depth sections from young bovine cartilage in order to investigate the depth dependence of collagen organization. The (*I*_2_, *φ*_2_) maps reproduce the well-established arcade structure of the fibrils as demonstrated in figure 3. The averaged orientation of collagen fibrils (*φ*_2_) follows a radial direction in the deep zone. This angle progressively realigns from perpendicular in the deep zone through the transitional zone, to parallel to the surface in the superficial zone. Interestingly, the molecular-scale order (*I*_2_) is lowest in the transitional zone, which can be attributed to a larger mixing of different fibrils directions, including out-of-plane for this specific geometry. In both the superficial and deep zone, the molecular-scale order parameter *I*_2_ indicates tight organization of collagen fibrils within the size of the focal spot (approx. 350 nm). The *I*_2_ values measured span between approximately 0.05 in the transition zone, to 0.2–0.3 in the superficial zone and 0.3–0.4 in the middle of the deep zone. These values can be translated into nonlinear tensorial ratio values, where the nonlinear ratio coefficient $\chi_{zzz}^{\left(2\right)}/\chi_{zxx}^{\left(2\right)}\;\;$is often used as a read-out, supposing the collagen arrangement of cylindrical symmetry with *z* the main fibril direction and *x* its perpendicular direction [32,36,41]. In particular values *I*_2_ between 0.1 and 0.4 correspond to $\chi_{zzz}^{\left(2\right)}/\chi_{zxx}^{\left(2\right)}\;\;$ratios between 1.2 and 1.65 (see electronic supplementary material 1). This can be compared to values typically measured in collagen type I in tendon where *I*_2_ ∼ 0.3 $\left(\chi_{zzz}^{\left(2\right)}/\chi_{zxx}^{\left(2\right)}\;=1.4\;\right)$ has been measured at the surface of rat tail tendon tissues (see electronic supplementary material 4).

**Figure 4.**
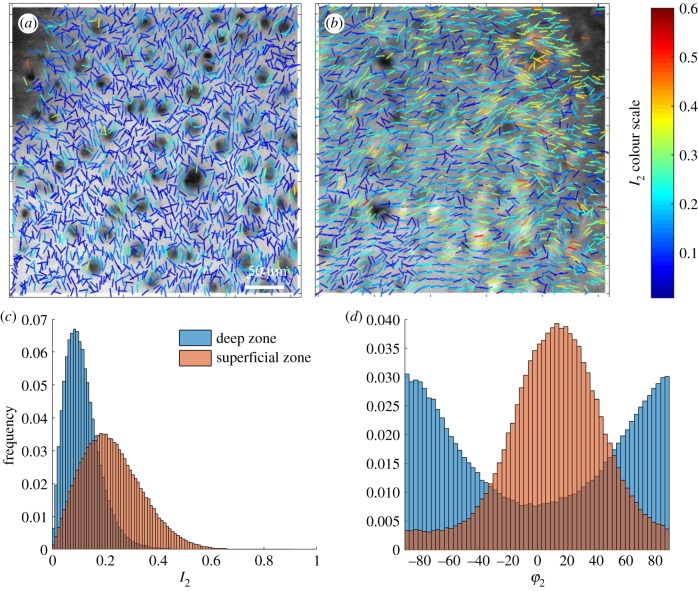
A comparison between deep zone (*a*) and superficial zone (*b*) collagen organization in sections cut parallel with the articular surface in the same specimen. (*a*,*b*) (*I*_2_, *φ*_2_) maps showing the collagen fibre orientations and degrees of order. (*c*) The distribution of *I*_2_ values over the superficial zone image and the deep zone image. (*d*) The distribution of *φ*_2_ over the deep zone and superficial images. (Online version in colour.)

In enface cartilage slices P-SHG measurements reveal differences in the 2D collagen organization between the deep and the superficial zone, where the collagen fibrils are mainly perpendicular to the image plane and parallel to the image plane respectively (figure 4*a*,*b*). As expected, the molecular-scale order values (*I*_2_) are very low in the deep zone (figure 4*c*), since the fibril distribution points perpendicularly to the sample plane where the polarization is rotated. The measured values are therefore underestimated since they are biased by their 2D projection in the polarization-rotation plane. Nevertheless, even in this deep zone where the collagen fibril alignment is predominantly perpendicular to the imaging plane there is still clearly a component of the fibril orientation within this plane and additionally when analysing the distribution of *φ*_2_ there is a preferred direction for this component of fibril orientation (figure 4*d*). As expected from the enface measurements (figure 4), the values of *I*_2_ are higher in the superficial zone (figure 4*b*) compared to the deep zone, with a more heterogeneous distribution of *I*_2_ in the superficial zone. The obtained averaged value at the superficial zone *I*_2_ ∼ 0.2 (corresponding to $\chi_{zzz}^{\left(2\right)}/\chi_{zxx}^{\left(2\right)}∼1.3$) reflects a less tight order of individual fibrils than in collagen I tendons (see electronic supplementary material 4) in agreement with values reported in the literature [41].

To investigate the organization of collagen as a function of depth, P-SHG stacks were taken. Example stacks for the deep and superficial zone are included in the electronic supplementary material (see electronic supplementary material 5). In the superficial zone, the values of *I*_2_ are highest at the articular surface, and then decrease with depth into the tissue, while in the deep zone *I*_2_ is constant with depth. In both zones, there is a slight rotation (16°–18°) in the angle *φ*_2_ with depth. Note that while *I*_2_ values can be used for quantitative analysis only when the collagen distribution lies in the sample plane, e.g. in the superficial zone, the angle *φ*_2_ always reports a reliable projection orientation of the collagen fibrils in the measurement plane.

**Figure 5.**
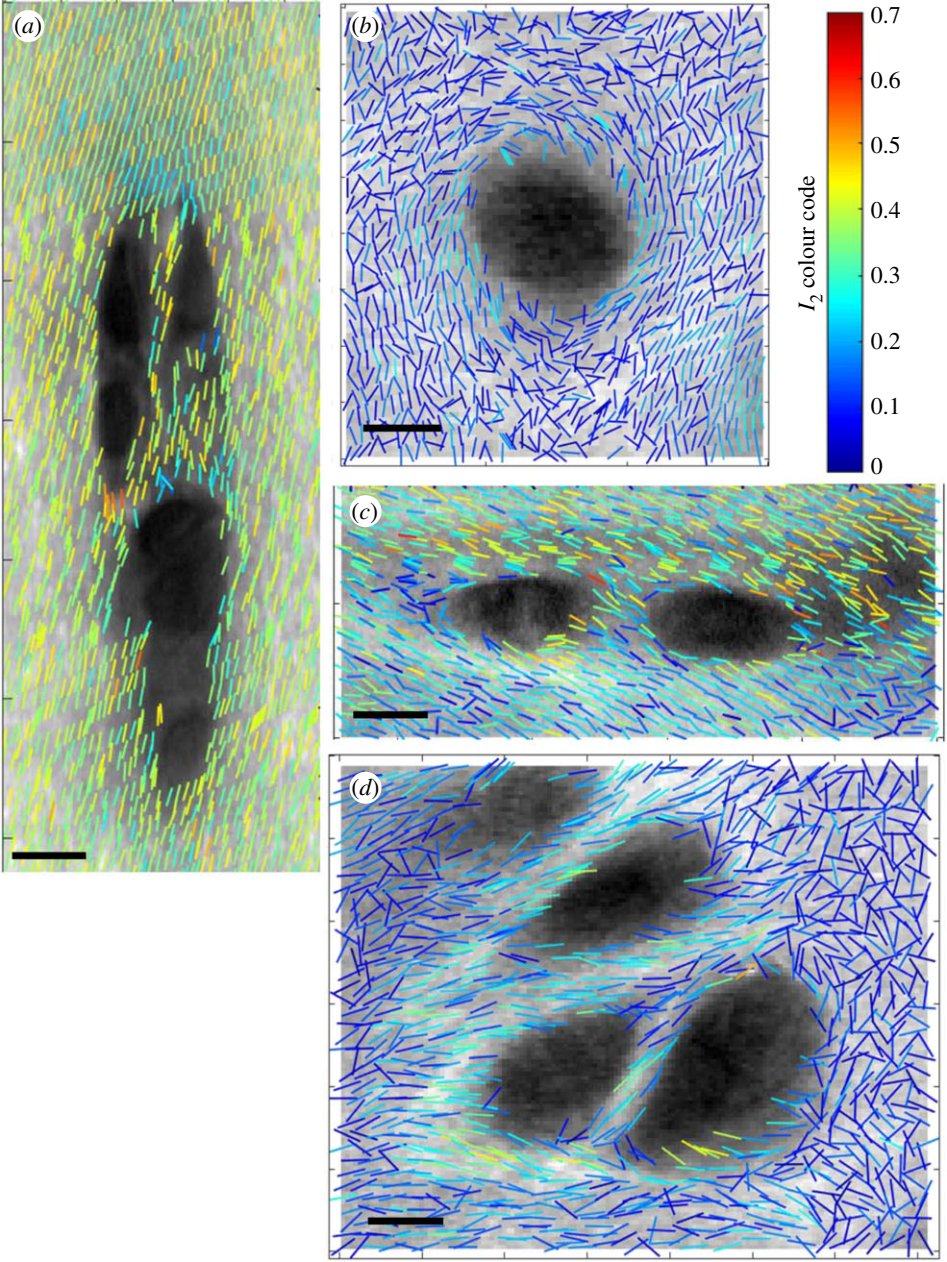
P-SHG around typical deep and superficial zone cells, with (*I*_2_, *φ*_2_) (*a*,*b*), deep zone cells in a transverse and enface section respectively, (*c*,*d*) superficial zone cells in transverse and enface sections. All scale bars are 10 µm. (Online version in colour.)

Figure 4 also shows variations in collagen organization in proximity to chondrocytes, in particular in the deep zone where the organization of collagen appears relatively more ordered, demonstrating in-plane re-organization of fibrils around the cells. Representative images of individual chondrocytes from the deep and superficial zone are shown in figure 5 (in this case *I*_2_ and *φ*_2_ are plotted at a higher density to increase resolution). In the columns of chondrocytes within the deep zone the collagen separating the cells is aligned radially (figure 5*a*). In the enface view of the deep zone chondrocyte there is some tangential alignment of the collagen (figure 5*b*). The pattern of alignment around groups of cells is more complex in the superficial zone, when imaged in the plane parallel to the articular surface (figure 5*d*), but most fibrils are roughly parallel to the articular surface (figure 5*c*).

**Figure 6.**
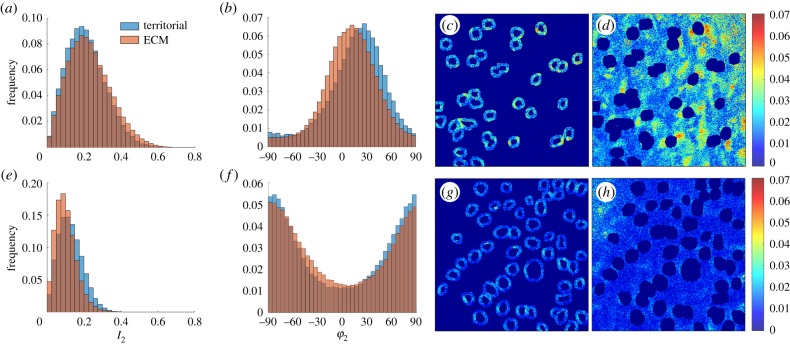
Comparison of collagen fibre organization in the territorial matrix and the bulk matrix. In this analysis, the territorial matrix is defined as a ring 5 µm thick surrounding the cells (*c*,*g*) and the bulk matrix is the rest of the extracellular matrix (*d*,*h*). Differences in the distribution of *I*_2_ are shown in panels (*a*) (surface zone) and (*e*) (deep zone) and *φ*_2_ in panels (*b*) (surface) and (*f*) (deep). (Online version in colour.)

The values of *I*_2_ and *φ*_2_ were compared for a 5 µm thick shell around the periphery of the cells, to those remote from the cells, for all en-face bovine samples imaged at zero strain (example images from one specimen are shown in figure 6). *I*_2_ is consistently greater in the territorial matrix of the deep zone chondrocytes compared to the rest of the extracellular matrix $\left(\Delta I_{2}=(I_{2(\mathrm{territorial})}-I_{2(\mathrm{bulk})})=0.022\pm 0.011,n=6\right)$, as also visible in figure 4. This shift in order is above the expected noise variations which lie close to 0.01. The differences in *I*_2_ between the territorial and extracellular matrix in the deep zone may reflect differences in the number of fibrils aligned in the image plane, with more fibrils in plane close to the cells. In the superficial zone, on the contrary, there is no significant difference $\left(\Delta I_{2}=0.006\pm 0.021,n=5\right)$. The distribution of *φ*_2_ also differs between the territorial and bulk matrix by up to 20°, but there is no consistent pattern.

### 3.2. Osteoarthritis cartilage

**Figure 7.**
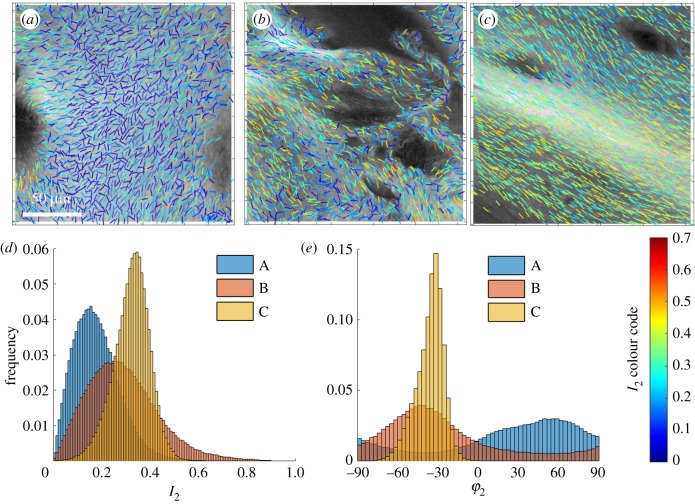
Osteoarthritic cartilage from total knee replacement surgery. (*a*–*c*) SHG images from different regions within a single section. (*d*) *I*_2_ distributions. (*e*) *φ*_2_ distributions. (Online version in colour.)

In OA, the changes in collagen organization are extensive, vary with the degree of degeneration and are heterogeneous on several length scales. This initial survey of submicron changes was undertaken on two samples from the lateral and medial femoral chondyle of patients undergoing knee replacement surgery with OA grading between 3 and 4 on the Outerbridge scale. Regions were selected in which there were no gross surface changes and full cartilage thickness was maintained. SHG shows a variety of patterns of collagen organization, often within a single section. Figure 7 illustrates this variability, with three regions selected from a total of 10 which were analysed to highlight the different structures that can be observed. Region *a* shows relatively low *I*_2_ values (0.18 ± 0.1) and a wide range of fibril angles, region *b* shows a highly disordered area with intermediate *I*_2_ values (0.29 ± 0.15), and region *c* shows highly parallel collagen fibrils with high *I*_2_ values (0.325 ± 0.07).

### 3.3. Collagen fibril reorganization with tensile load

**Figure 8.**
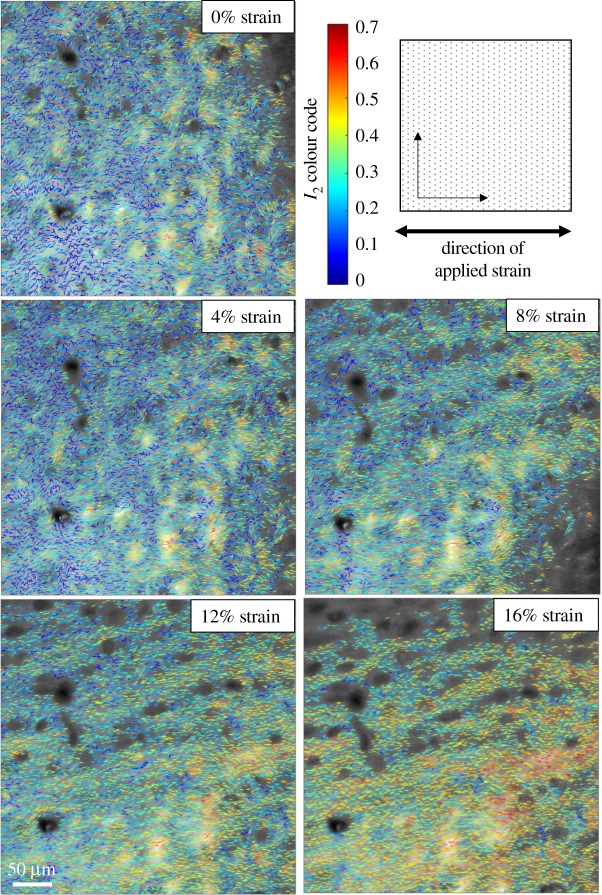
(*I*_2_, *φ*_2_) maps of a specimen of superficial zone bovine cartilage as strain is increased along the *x*-axis from 0% to 16%. (Online version in colour.)

Figure 8 shows representative results in the superficial zone of a specimen exposed to steps of strain up to 16%. As the strain increases *φ*_2_ rotates towards the direction of applied strain (*x*-axis) and the value of *I*_2_ increases (as shown by an increase in the number of red and yellow lines in the colour coded image).

**Figure 9.**
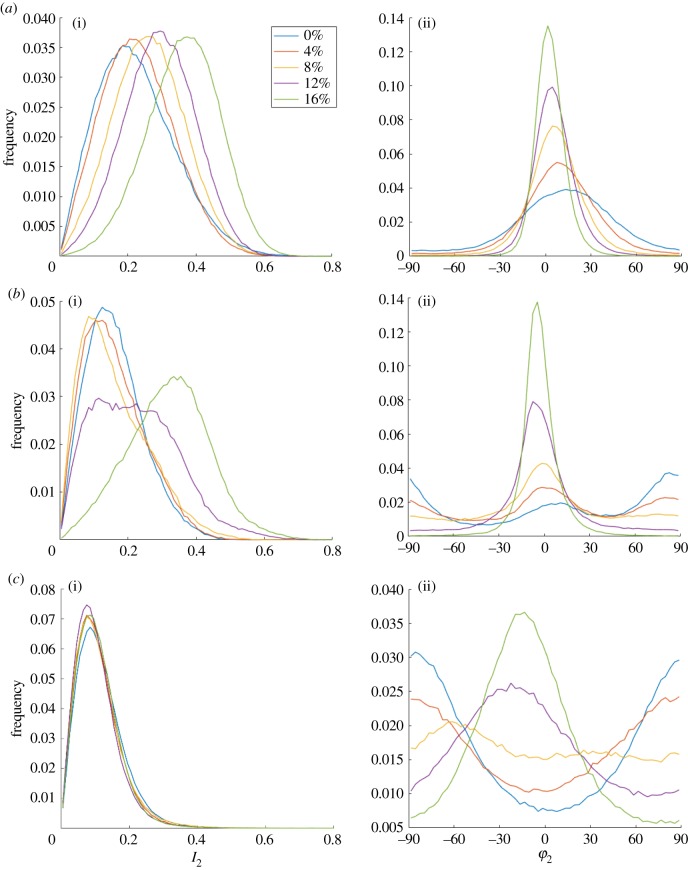
Changes in *I*_2_ and *φ*_2_ with strain. (*a*) Sample from the superficial zone as shown in figure 8, initial fibre orientation parallel to applied strain. (*b*) Superficial zone, initial fibre orientation perpendicular to applied strain. (*c*) Deep zone, initial fibre direction perpendicular to applied strain. (*φ*_2_ = 0° is parallel to the direction of applied strain.) (Online version in colour.)

Eleven samples were tensile loaded (five from the superficial zone, three from the deep zone of bovine cartilage and three from human OA cartilage). The P-SHG changes vary between cartilage zones and also depend on the initial orientation of fibrils with respect to the applied strain. Figure 9 shows the changes in three specimens which illustrate most of the behaviour noted throughout the whole group. Figure 9*a* shows the sample used in figure 8, in which the preferred collagen direction is initially approximately parallel with the applied strain. As the strain increases the mean *φ*_2_ moves towards 0° and the distribution of fibril angles becomes tighter: at the same time the values of *I*_2_ increase. Figure 9*b* shows a specimen from the superficial zone with a bimodal fibril distribution in the relaxed state with peaks at 15° and 80°. As the strain increases from 0 to 8% the peak at 80° is lost and the other peak shifts towards 0°, with a slight decrease in *I*_2_. At strains between 8 and 12% *I*_2_ then starts to increase more rapidly and *φ*_2_ becomes more tightly centred around the direction of applied strain. Lastly, figure 9*c* shows a sample from the deep zone in which *φ*_2_ is initially distributed around a peak perpendicular to the direction of applied strain and as the strain increases this peak disappears and a new peak of similar width emerges parallel to the direction of applied strain and there is no increase in *I*_2_ with strain.

**Figure 10.**
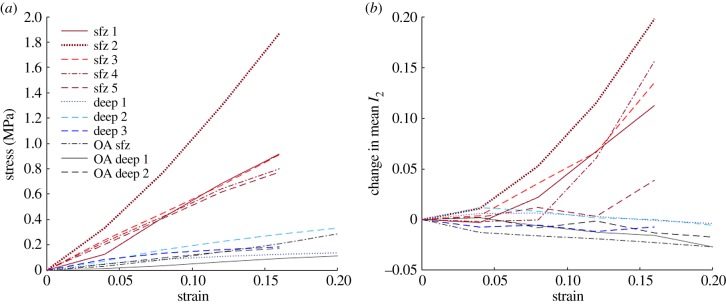
(*a*) Stress–strain curves from all tensile tested specimens. (*b*) Change in *I*_2_ as a function of strain. (Line colours and hatchings are matched between images—red (Sfz1–5) = superficial zone bovine, blue (deep 1–3) = deep zone bovine, grey (OA1–3) = human.) (Online version in colour.)

In order to establish the relationship between macroscopic strain applied to cartilage and the sub-micron response of the collagen fibrils we compared the changes in *I*_2_ and *φ*_2_ to the stress–strain curves for each cartilage specimen (figure 10). The macroscopic stress–strain curves show the expected pattern, tensile moduli in the surface zone being 2.5–11-fold higher than those in the deep zone [5]. In the superficial zone *I*_2_ increases with strain and the magnitude of the change in the five bovine samples is greater the higher the elastic modulus, but in the deep zone there is no significant change in *I*_2_ with strain. In the superficial zone only there is a decrease in the spread of *φ*_2_ as illustrated in figures 9*a*,*b*.

**Figure 11.**
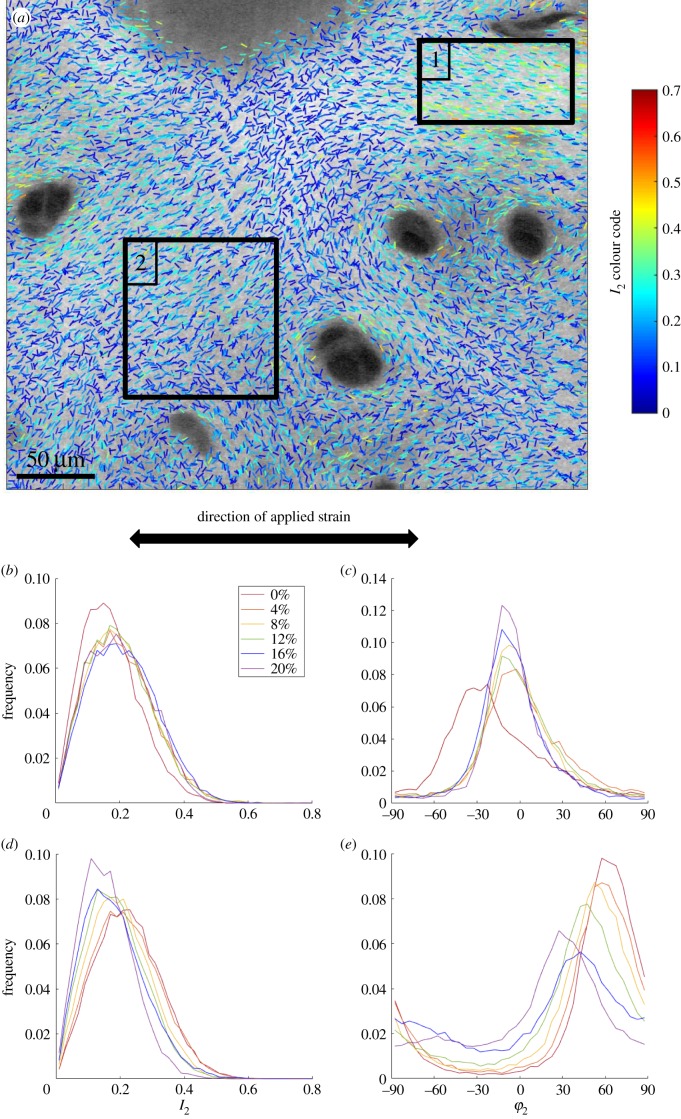
(*a*) P-SHG image of a sample of OA cartilage at zero strain. Regions 1 and 2 were selected for analysis of changes with strain, applied in the direction of the arrow. In region 1 the initial *φ*_2_ peak is 30° and panels (*b*) and (*c*) show changes in *I*_2_ and *φ*_2_. In region 2 the initial *φ*_2_ peak is 70° and panels (*d*) and (*e*) the changes in *I*_2_ and *φ*_2_. (Online version in colour.)

Finally, we investigated the behaviour of osteoarthritic cartilage under strain. The stress–strain curves are shown in figure 10 and indicate lower strain moduli than bovine cartilage, particularly in the superficial zone. These differences are consistent with our own observations on a larger group of specimens (J Mansfield, V Mandalia, A Toms, CP Winlove 2017, unpublished data) and other studies [4,6,7]. However, the strain fields in osteoarthritic tissue are extremely heterogeneous within each field of view and different regions show different patterns of fibril re-orientation as indicated in figure 11. Here in region 1, selected as showing higher *I*_2_ and initial fibril angles around 30° the fibrils realign towards the direction of applied strain with a modest increase in *I*_2_. In region 2, approximately 120 µm away, the preferred direction of *φ*_2_ is initially approximately 70° to the applied strain and as the strain increases it slowly rotates towards the direction of applied strain, while *I*_2_ actually decreases with strain.

## 4. Discussion

The diameter of collagen fibrils is below the diffraction limit for light microscopy and polarized light microscopy has, since Bennighoff's classical description of the collagen architecture, been a powerful tool for cartilage research [15], although for quantitative analysis it is restricted to specimens of known optical thickness. More recently SHG, exploiting symmetry breaking within the fibril structure, provides label free visualization of collagen fibrils and has paved the way for numerous studies of the role of collagen fibrils in tissue mechanics [8,9,47,48]. Analysis of the polarization of the SHG signal should provide further insights into the structure of type II collagen and its changes with mechanical load and disease.

In interpreting our observations, it is important to consider the scale of the measurements. The type II collagen fibril is a hierarchical structure with molecules 300 × 2 nm stacked laterally and longitudinally into arrays 20–100 nm in diameter and without detectable ends in cartilage. The SHG signal is presumed to arise from dipoles associated with the regular peptide bond structure of the collagen molecule. In our current system, the P-SHG for each pixel arises from a volume approximately 0.1 fl and therefore will be an average value from multiple fibrils transecting this volume. Accessing finer scales is possible only using non-optical methods such as X-ray scattering, which are difficult both to implement and analyse.

The scale of our measurements lies between those of conventional light microscopy and electron microscopy. TEM remains one of the best ways to visualize individual fibrils in cartilage, however due to the requirement for very thin slices and the small fields of view it is less suitable for mapping larger scale variations in fibril architecture. Additionally in mechanical loading experiments it can only provide a snap shot of fibril organizations [49] whereas P-SHG allows us to track changes in the collagen fibril distribution at the molecular scale. Detailed insights into the intrafibrillar structure of collagen and its response to mechanical loads on even smaller scales have emerged from studies on the thicker fibres of type I collagen which have used a combination of wide and small angle X-ray scattering to determine molecular packing and alignment within fibres [50,51]. X-ray scattering has also been used to investigate cartilage collagen [52–55] including how this changes under compressive loads [53,54]. Most recently, this approach has revealed differences in the d-spacing with depth [53], which might be related to the differences we observed in the present study. Further work might exploit the complementarity between the two approaches.

The present work reveals aspects of collagen organization that can be related to previous studies in electron microscopy. Our observations show a visibly tighter collagen alignment along the deep zone as compared to the transition and even superficial zone, which resembles bundled structures observed in this region using EM [56]. We also observe a clear re-orientation of collagen fibrils along the diameter of the chondrocytes, with tighter sub-micrometric scale organization in a direction perpendicular to the main collagen direction of the deep zone. Electron microscopy of the collagen in the pericellular matrix of chondrocytes shows a basket-like structure around the cells [57]. Collagen polarity has been also observed to vary strongly in the proximity of these regions [42], which is consistent with this more complex organization. The true pericellular matrix could not be resolved in the present work, and whether this was because of collagens such as type VI are weak generators of SHG or because of the geometrical complexity of the cell boundary must be established in future work. However, P-SHG showed that the direction of the collagen was disturbed around the cells over rather larger distances and the biomechanical implications of these variations will require consideration in tracking the exchange of mechanical signals between chondrocytes and the bulk matrix.

P-SHG provides a new perspective on the response of the collagen fibrils to mechanical load. As might be expected, as the strain increases the average value of *φ*_2_ aligns towards the direction of applied strain. However, an interesting situation arises for fibrils perpendicular to the direction of applied load, because two scenarios can be envisioned: (i) the peak *φ*_2_ progressively shifts towards the direction of applied strain but remains approximately constant in height; this cooperative behaviour would be indicative of tissue reorganization on a length scale greater than the field of view or (ii) the peak in *φ*_2_ perpendicular to the strain direction decreases in intensity and a separate peak in *φ*_2_ emerges parallel to the direction of applied strain, indicating re-organization on a shorter length scale. In figure 9*b* and *c* the latter pattern was prevalent. Unravelling the processes of mechanotransduction in a cell interacting with fibrils of different initial orientations is a challenge for the future.

More generally, in exploring correlations between the macroscopic mechanical properties of the tissue and the micro-structural behaviour of the collagen fibrils it is important to recognize that P-SHG provides information on two length scales. Changes in *φ*_2_ reflect the reorientation of collagen fibrils on a relatively large scale, as discussed above, and these data could provide inputs for structurally based finite element models relating tissue mechanics to fibril organization [58]. In addition, changes in *I*_2_ indicate changes in fibril structure on the submicron length scale. The most significant changes in *I*_2_ were observed in the superficial zone, however this is mostly attributed to the limitation of the technique is that P-SHG can only measure the organization of fibrils aligned in the image plane. In the deep zone this was, with the geometry employed in the present investigation, true for only a minority of fibrils, although these were the ones bearing the applied load. Figure 10 shows that, in the small number of samples studied, there is a rough correlation between the increase in *I*_2_ under tensile loading and the tensile modulus of the sample, particularly in the superficial zone, perhaps indicating that higher forces are required to generate these changes. Recent work on X-ray scattering in cartilage has shown changes in d-spacing in collagen fibrils under mechanical load [53]. Future work might establish the relationships between these observations, but it is clear that mechanical forces are transmitted down to the lowest levels of collagen structure.

The changes to the collagen matrix, which occur in OA, are complicated and include softening (loosening of the collagen II matrix), fibrillation and replacement of the type II collagen with type I collagen. The small number of osteoarthritic cartilage samples included in this study were representative of the spectrum of changes reported in the literature [10] and we found them to be they associated with fibril re-organization on a sub-micron scale, reflected in both *I*_2_ and *φ*_2_. Whether these small scale changes are consequences of the larger-scale changes, for example a release in fibril tension following alterations in the overall force balance in the tissue, or indicate the effects of specific processes, such as matrix proteolysis, that initiate the macroscopic changes of OA remains to be established.

## 5. Conclusion

This paper reports techniques of P-SHG that are directly applicable to investigating the microstructural response to mechanical loading in collagen-rich tissues. We demonstrate regional and local patterns in the sub-micron scale organization of articular collagen and that this organization is responsive to changes in mechanical loading. A more extensive study on the polarization properties of degenerate and ageing cartilage collagen should reveal the mechanisms of sub-micron scale remodelling and their role in the progression of disease. This may further demonstrate the potential of P-SHG as a diagnostic tool for assessing cartilage quality in early OA.

## Supplementary Material

supplementary materials 1-4

## Supplementary Material

supplementary materials 5

## Data accessibility

The raw image and load cell data collected for this publication are available at https://doi.org/10.24378/exe.603 along with the MATLAB code required to perform the polarization sensitivity analysis.

## References

[1] Stockwell RA 1979 Biology of cartilage cells. Cambridge, UK: Cambridge University Press.

[2] Eyre D 2002 Collagen of articular cartilage. Arthritis Res. 4, 30–35. (10.1186/ar380) 11879535 PMC128915

[3] Huang CY, Stankiewicz A, Ateshian GA, Mow VC 2005 Anisotropy, inhomogeneity, and tension–compression nonlinearity of human glenohumeral cartilage in finite deformation. J. Biomech. 38, 799–809. (10.1016/j.jbiomech.2004.05.006) 15713301 PMC3786419

[4] Kempson G, Freeman M, Swanson S 1968 Tensile properties of articular cartilage. Nature 220, 1127–1128. (10.1038/2201127b0) 5723609

[5] Woo SLY, Akeson W, Jemmott G 1976 Measurements of nonhomogeneous, directional mechanical properties of articular cartilage in tension. J. Biomech. 9, 785–791. (10.1016/0021-9290(76)90186-X) 1022791

[6] Akizuki S, Mow V, Müller F, Pita J, Howell D, Manicourt D 1986 Tensile properties of human knee joint cartilage: I. Influence of ionic conditions, weight bearing, and fibrillation on the tensile modulus. J. Orthop. Res. 4, 379–392. (10.1002/jor.1100040401) 3783297

[7] Kempson G 1982 Relationship between the tensile properties of articular cartilage from the human knee and age. Ann. Rheum. Dis. 41, 508–511. 7125720 10.1136/ard.41.5.508 PMC1001032

[8] Bell JS, Christmas J, Mansfield JC, Everson RM, Winlove CP 2014 Micromechanical response of articular cartilage to tensile load measured using nonlinear microscopy. Acta Biomater. 10, 2574–2581. (10.1016/j.actbio.2014.02.008) 24525036

[9] Mansfield JC, Bell JS, Winlove CP 2015 The micromechanics of the superficial zone of articular cartilage. Osteoarthritis Cartilage 23, 1806–1816. 26050867 10.1016/j.joca.2015.05.030

[10] Chen MH, Broom N 1998 On the ultrastructure of softened cartilage: a possible model for structural transformation. J. Anat. 192, 329–341. (10.1046/j.1469-7580.1998.19230329.x) 9688499 PMC1467777

[11] Chen MH, Broom ND 1999 Concerning the ultrastructural origin of large-scale swelling in articular cartilage. J. Anat. 194, 445–461. (10.1046/j.1469-7580.1999.19430445.x) 10386781 PMC1467943

[12] Jeffery AK, Blunn GW, Archer CW, Bentley G 1991 3-Dimensional collagen architecture in bovine articular-cartilage. J. Bone Joint Surg. Br. 73, 795–801. (10.1302/0301-620X.73B5.1894669) 1894669

[13] Poole CA, Flint MH, Beaumont BW 1987 Chondrons in cartilage—ultrastructural analysis of the pericellular microenvironment in adult human articular cartilages. J. Orthop. Res. 5, 509–522. (10.1002/jor.1100050406) 3681525

[14] Nieminen M, Rieppo J, Töyräs J, Hakumäki JM, Silvennoinen J, Hyttinen MM, Helminen HJ, Jurvelin JS 2001 *T*_2_ relaxation reveals spatial collagen architecture in articular cartilage: a comparative quantitative MRI and polarized light microscopic study. Magn. Reson. Med. 46, 487–493. (10.1002/mrm.1218) 11550240

[15] Rieppo J, Hallikainen J, Jurvelin JS, Kiviranta I, Helminen HJ, Hyttinen MM 2008 Practical considerations in the use of polarized light microscopy in the analysis of the collagen network in articular cartilage. Microsc. Res. Tech. 71, 279–287. (10.1002/jemt.20551) 18072283

[16] Drexler W et al. 2001 Correlation of collagen organization with polarization sensitive imaging of *in vitro* cartilage: implications for osteoarthritis. J. Rheumatol. 28, 1311–1318. 11409125

[17] Ugryumova N, Jacobs J, Bonesi M, Matcher SJ 2009 Novel optical imaging technique to determine the 3-D orientation of collagen fibers in cartilage: variable-incidence angle polarization-sensitive optical coherence tomography. Osteoarthritis Cartilage 17, 33–42. (10.1016/j.joca.2008.05.005) 18621555

[18] Chen X, Nadiarynkh O, Plotnikov S, Campagnola PJ 2012 Second harmonic generation microscopy for quantitative analysis of collagen fibrillar structure. Nat. Protoc. 7, 654 (10.1038/nprot.2012.009) 22402635 PMC4337962

[19] Zipfel WR, Williams RM, Christie R, Nikitin AY, Hyman BT, Webb WW 2003 Live tissue intrinsic emission microscopy using multiphoton-excited native fluorescence and second harmonic generation. Proc. Natl Acad. Sci. USA 100, 7075–7080. (10.1073/pnas.0832308100) 12756303 PMC165832

[20] Tilbury KB, Hocker JD, Wen BL, Sandbo N, Singh V, Campagnola PJ 2014 Second harmonic generation microscopy analysis of extracellular matrix changes in human idiopathic pulmonary fibrosis. *J. Biomed. Optics* **15**, 086014. 10.1117/1.JBO.19.8.086014 PMC4137064 25134793

[21] Ghazaryan A, Tsai HF, Hayrapetyan G, Chen W-L, Chen Y-F, Jeong M-Y, Kim C-S, Chen S-J, Dong C-Y 2012 Analysis of collagen fiber domain organization by Fourier second harmonic generation microscopy. *J. Biomed. Optics* **18**, 031105.

[22] Mostaço-Guidolin LB, Ko ACT, Wang F, Xiang B, Hewko M, Tian G, Major A, Shiomi M, Sowa MG 2013 Collagen morphology and texture analysis: from statistics to classification. Sci. Rep. 3, 2190 (10.1038/srep02190) 23846580 PMC3709165

[23] Chaudhary R, Campbell KR, Tilbury KB, Vanderby R, Block WF, Kijowski R, Campagnola PJ 2015 Articular cartilage zonal differentiation via 3D second-harmonic generation imaging microscopy. Connect. Tissue Res. 56, 76–86. (10.3109/03008207.2015.1013192) 25738523 PMC4497507

[24] Brown CP, Houle MA, Chen M, Price AJ, Legare F, Gill HS 2012 Damage initiation and progression in the cartilage surface probed by nonlinear optical microscopy. J. Mech. Behav. Biomed. Mater. 5, 62–70. (10.1016/j.jmbbm.2011.08.004) 22100080

[25] Freund I, Deutsch M, Sprecher A 1986 Connective-tissue polarity—optical 2nd-harmonic microscopy, crossed-beam summation, and small-angle scattering in rat-tail tendon. Biophys. J. 50, 693–712. (10.1016/S0006-3495(86)83510-X) 3779007 PMC1329848

[26] Stoller P, Kim BM, Rubenchik AM, Reiser KM, Da Silva LB 2002 Polarization-dependent optical second-harmonic imaging of a rat-tail tendon. J. Biomed. Opt. 7, 205–214. (10.1117/1.1431967) 11966305

[27] Williams RM, Zipfel WR, Webb WW 2005 Interpreting second-harmonic generation images of collagen I fibrils. Biophys. J. 88, 1377–1386. (10.1529/biophysj.104.047308) 15533922 PMC1305140

[28] Aït-Belkacem D, Gasecka A, Munhoz F, Brustlein S, Brasselet S 2010 Influence of birefringence on polarization resolved nonlinear microscopy and collagen SHG structural imaging. Opt. Express 18, 14 859–14 870. (10.1364/OE.18.014859) 20639973

[29] Brasselet S 2011 Polarization-resolved nonlinear microscopy: application to structural molecular and biological imaging. Adv. Opt. Photonics 3, 205 (10.1364/AOP.3.000205)

[30] Duboisset J, Aït-Belkacem D, Roche M, Rigneault H, Brasselet S 2012 Generic model of the molecular orientational distribution probed by polarization-resolved second-harmonic generation. Phys. Rev. A 85, 043829 (10.1103/PhysRevA.85.043829)

[31] Tiaho F, Recher G, Rouede D 2007 Estimation of helical angles of myosin and collagen by second harmonic generation imaging microscopy. Opt. Express 15, 12 286–12 295. (10.1364/OE.15.012286) 19547597

[32] Psilodimitrakopoulos S, Artigas D, Soria G, Amat-Roldan I, Planas AM, Loza-Alvarez P 2009 Quantitative discrimination between endogenous SHG sources in mammalian tissue, based on their polarization response. Opt. Express 17, 10 168–10 176. (10.1364/OE.17.010168) 19506670

[33] Chen WL, Li TH, Su PJ, Chou CK, Fwu PT, Lin SJ, Kim D, So PTC, Dong CY 2009 Second harmonic generation chi tensor microscopy for tissue imaging. Appl. Phys. Lett. 94, 183902 (10.1063/1.3132062)

[34] Tilbury K, Lien C-H, Chen S-J, Campagnola PJ 2014 Differentiation of Col I and Col III isoforms in stromal models of ovarian cancer by analysis of second harmonic generation polarization and emission directionality. Biophys. J. 106, 354–365. (10.1016/j.bpj.2013.10.044) 24461010 PMC3907237

[35] Stoller P, Reiser KM, Celliers PM, Rubenchik AM 2002 Polarization-modulated second harmonic generation in collagen. Biophys. J. 82, 3330–3342. (10.1016/S0006-3495(02)75673-7) 12023255 PMC1302120

[36] Kumar R, Grønhaug KM, Romijn EI, Finnøy A, Davies CL, Drogset JO, Lilledahl MB 2015 Polarization second harmonic generation microscopy provides quantitative enhanced molecular specificity for tissue diagnostics. J. Biophotonics 8, 730–739. (10.1002/jbio.201400086) 25363416

[37] Aït-Belkacem D, Roche M, Duboisset J, Ferrand P, Brasselet S, Guilbert M, Sockalingum GD, Jeannesson P 2012 Microscopic structural study of collagen aging in isolated fibrils using polarized second harmonic generation. *J. Biomed. Optics* **17**, 080506. 10.1117/1.JBO.17.8.080506 23224157

[38] Strupler M, Hernest M, Fligny C, Martin J-L, Tharaux P-L, Schanne-Klein M-C 2008 Second harmonic microscopy to quantify renal interstitial fibrosis and arterial remodeling. J. Biomed. Opt. 13, 054041 (10.1117/1.2981830) 19021421

[39] Ambekar R, Lau T-Y, Walsh M, Bhargava R, Toussaint KC 2012 Quantifying collagen structure in breast biopsies using second-harmonic generation imaging. Biomed. Opt. Express 3, 2021–2035. (10.1364/BOE.3.002021) 23024898 PMC3447546

[40] Gusachenko I, Tran V, Houssen YG, Allain JM, Schanne-Klein MC 2012 Polarization-resolved second-harmonic generation in tendon upon mechanical stretching. Biophys. J. 102, 2220–2229. (10.1016/j.bpj.2012.03.068) 22824287 PMC3341536

[41] Su PJ et al. 2010 The discrimination of type I and type II collagen and the label-free imaging of engineered cartilage tissue. Biomaterials 31, 9415–9421. (10.1016/j.biomaterials.2010.08.055) 20875682

[42] Couture C-A et al. 2015 The impact of collagen fibril polarity on second harmonic generation microscopy. Biophys. J. 109, 2501–2510. (10.1016/j.bpj.2015.10.040) 26682809 PMC4699883

[43] Outerbridge RE 1961 The etiology of chondromalacia patellae. J. Bone Joint Surg. Br. 43, 752–757. (10.1302/0301-620X.43B4.752) 14038135

[44] Deniset-Besseau A, Duboisset J, Benichou E, Hache F, Brevet P-F, Schanne-Klein M-C 2009 Measurement of the second-order hyperpolarizability of the collagen triple helix and determination of its physical origin. J. Phys. Chem. B 113, 13 437–13 445. (10.1021/jp9046837) 19754079

[45] Schon P, Behrndt M, Ait-Belkacem D, Rigneault H, Brasselet S 2010 Polarization and phase pulse shaping applied to structural contrast in nonlinear microscopy imaging. Phys. Rev. A 81, 013809 (10.1103/PhysRevA.81.013809)

[46] Benninghoff A 1925 Form und bau der Gelenknorpel in ihren Bezeihungen zur Function. Z Zellforsch Mikrosk Anatomy 2, 783–825. (10.1007/BF00583443)

[47] Alkhouli N et al. 2013 The mechanical properties of human adipose tissues and their relationships to the structure and composition of the extracellular matrix. Am. J. Physiol. Endocrinol. Metab. 305, E1427–E1435. 24105412 10.1152/ajpendo.00111.2013

[48] Vergari C, Mansfield J, Meakin JR, Winlove PC 2016 Lamellar and fibre bundle mechanics of the annulus fibrosus in bovine intervertebral disc. Acta Biomater. 37, 14–20. (10.1016/j.actbio.2016.04.002) 27063647

[49] Sasazaki Y, Shore R, Seedhom BB 2006 Deformation and failure of cartilage in the tensile mode. J. Anat. 208, 681–694. (10.1111/j.1469-7580.2006.00569.x) 16761971 PMC2100232

[50] Bell JS et al. 2018 The hierarchical response of human corneal collagen to load. Acta Biomater. 65, 216–225. (10.1016/j.actbio.2017.11.015) 29128531 PMC5729024

[51] Fratzl P, Daxer A 1993 Structural transformation of collagen fibrils in corneal stroma during drying. An X-ray scattering study. Biophys. J. 64, 1210–1214. (10.1016/S0006-3495(93)81487-5) 8494978 PMC1262438

[52] Moger CJ, Barrett R, Bleuet P, Bradley DA, Ellis RE, Green EM, Knapp K, M P, Winlove CP 2007 Regional variations of collagen orientation in normal and diseased articular cartilage and subchondral bone determined using small angle X-ray scattering (SAXS). Osteoarthritis Cartilage 15, 682–687. (10.1016/j.joca.2006.12.006) 17306566

[53] Inamdar SR, Knight DP, Terrill NJ, Karunaratne A, Cacho-Nerin F, Knight MM, Gupta HS 2017 The secret life of collagen: temporal changes in nanoscale fibrillar pre-strain and molecular organization during physiological loading of cartilage. ACS Nano 11, 9728–9737. (10.1021/acsnano.7b00563) 28800220

[54] Moger CJ, Arkill KP, Barrett R, Bleuet P, Ellis RE, Green EM, Winlove CP 2009 Cartilage collagen matrix reorientation and displacement in response to surface loading. J. Biomech. Eng. 131, 031008 (10.1115/1.3049478) 19154067

[55] Aspden RM, Hukins DW 1981 Collagen organization in articular cartilage, determined by X-ray diffraction, and its relationship to tissue function. Proc. R. Soc. Lond. B 212, 299–304. 6115394 10.1098/rspb.1981.0040

[56] Hughes L, Archer C, Ap Gwynn I 2005 The ultrastructure of mouse articular cartilage: collagen orientation and implications for tissue functionality. A polarised light and scanning electron microscope study and review. Eur. Cell. Mater. 9, 68–84. (10.22203/eCM.v009a09) 15968593

[57] Poole CA 1997 Articular cartilage chondrons: form, function and failure. J. Anat. 191, 1–13. (10.1046/j.1469-7580.1997.19110001.x) 9279653 PMC1467653

[58] Julkunen P, Kiviranta P, Wilson W, Jurvelin JS, Korhonen RK 2007 Characterization of articular cartilage by combining microscopic analysis with a fibril-reinforced finite-element model. J. Biomech. 40, 1862–1870. (10.1016/j.jbiomech.2006.07.026) 17052722

