# Supplementary material for: Collagen reorganization in cartilage under strain probed by polarization sensitive second harmonic generation microscopy
Source: J R Soc Interface. 2019 Jan 16;16(150):20180611. doi: 10.1098/rsif.2018.0611 (PMC6364654; doi:10.1098/rsif.2018.0611)
Supplement: supplementary materials 1-4 [file rsif20180611supp1.docx]

**Supplementary Materials 1. Relation between nonlinear induced dipoles distributions, measured anisotropy** $\boldsymbol{I}_{\boldsymbol{2}}$**parameter, and nonlinear tensor characteristics in collagen.**

The polarization dependence of the SHG intensity can be deduced from a theoretical distribution of nonlinear induced dipoles, supposing a coherent superposition of all dipoles in the focal spot of the excitation objective. Under the dipolar approximation that holds within the size of the diffraction limit size, each nonlinear contribution can be summed up coherently. SHG from a single molecule (or peptide bond in the case of collagen) is the result of the radiation from the induced individual dipole $\boldsymbol{p}_{SHG}\propto\beta\left( \Omega\right):\boldsymbol{E}\left( \alpha\right)\boldsymbol{E}\left( \alpha\right)$, where $\beta$ is the nonlinear susceptibility tensor of the single molecule and $\boldsymbol{E}$ is the incident field at the frequency $\omega$, linearly polarized along an angle $\alpha$ with respect to$X$ if $(X,Y)$ is the sample plane. At the ensemble level, these dipoles radiate coherently within the focal volume to lead to a macroscopic nonlinear induced dipole [1, 2]:

$\boldsymbol{P}_{SHG}\propto N\int\left[ \beta\left( \Omega\right):\boldsymbol{E}\left( \alpha\right)\boldsymbol{E}\left( \alpha\right) \right] f\left( \Omega\right)d\Omega=\chi^{(2)}:\boldsymbol{E}\left( \alpha\right)\boldsymbol{E}\left( \alpha\right)$

with $N$ the molecular density and $f\left( \Omega\right)$ the molecular angular distribution of individual nonlinear dipoles in the excitation focal spot, with $\Omega=(\theta,\varphi)$ their orientation in the macroscopic frame $(X,Y,Z)$ . The macroscopic nonlinear tensor representative of the molecular ensemble present in the focal volume thus take the form:

$$\chi^{(2)}=N\int\beta\left( \Omega\right) f\left( \Omega\right)d\Omega$$

The measured SHG intensity can be deduced from the resulting radiated field: $\boldsymbol{E}_{SHG}\propto\boldsymbol{k}\times\boldsymbol{k}\times\boldsymbol{P}_{SHG}$, which leads to, in a paraxial approximation: $\boldsymbol{I}_{SHG}\propto\left| \boldsymbol{P}_{SHG} \right|^{2}$. From its fourth power dependence in $\boldsymbol{E}\left( \alpha\right)=(\cos\alpha,\sin\alpha,0)$, this intensity can thus be decomposed in contributions of different harmonics in $\alpha:$

$$I_{SHG}\left( \alpha\right)\propto a_{0}+a_{2}\cos2\alpha+b_{2}\sin2\alpha{+a}_{4}\cos4\alpha+b_{4}\sin4\alpha$$

The coefficients can be grouped into amplitude and phase coefficients of different orders of symmetry:

$I_{SHG}\left( \alpha\right)\propto a_{0}+I_{2}\cos2(\alpha-\varphi_{2})+I_{4}\cos4(\alpha-\varphi_{4})$

With:

$I_{2}= \frac{\sqrt{a_{2}^{2}+b_{2}^{2}}}{a_{0}} , I_{4}= \frac{\sqrt{a_{4}^{2}+b_{4}^{2}}}{a_{0}} ,$ $\varphi_{2}=0.5 \tan^{-1} \left( \frac{b_{2}}{a_{2}} \right), \varphi_{4}={0.25 tan}^{-1} \left( \frac{b_{4}}{a_{4}} \right)$

The second order parameters $(I_{2},\varphi_{2})$ represent the magnitude and orientation of the anisotropic contribution to the polarization response $I\left( \alpha\right)$, while $(I_{4},\varphi_{4})$ are the magnitude and orientation signatures of its more complex fourth order dependence.

In distributions of cylindrical symmetry, such as expected in a collection of collagen fibres aligned along a main direction $\varphi_{2}$, the fourth order dependence of $I\left( \alpha\right)$ is aligned along its second order dependence, leading to $\varphi_{2}=\varphi_{4}$. Any deviation to this symmetry is likely to lead to a dissymmetric polarization response, whose fourth order can be decomposed into an antisymmetric and a symmetric contribution [3]:

$$I_{4a}=I_{4}\sin\left( \varphi_{4}-\varphi_{2} \right)$$

$$I_{4s}=I_{4}\cos\left( \varphi_{4}-\varphi_{2} \right)$$

The ${(I}_{2},I_{4})$ coefficients can be related to the way molecules are organized in a sample. In what follows, we detail the relation between these coefficients and the parameters that characterize the organization of collagen fibres present in the focal volume: (1) the macroscopic tensor $\chi^{(2)}$ formed by the structure and (2) the angular extent of the distribution of nonlinear induced molecular-scale dipoles.

1. *Relation to the macroscopic tensor* $\chi^{(2)}$. The P-SHG intensity is related to this tensor by the relation

$$\boldsymbol{I}_{SHG}\left( \alpha\right)\propto\left| \chi^{\left( 2 \right)}:\boldsymbol{E}\left( \alpha\right)\boldsymbol{E}\left( \alpha\right) \right|^{2}=\sum_{I,J,K=X,Y} \chi_{IJK}^{\left( 2 \right)}{\left( \rho,\eta\right)E}_{J}E_{K}$$

With $\left( \rho,\eta\right)$ the orientation of the collagen fibre in the macroscopic frame, and $\chi_{IJK}^{\left( 2 \right)}\left( \rho,\eta\right)=\sum_{i,i,k=x,y,z} \chi_{ijk}^{\left( 2 \right)}\left( \boldsymbol{I.i} \right)\left( \boldsymbol{J.j} \right)\left( \boldsymbol{K.k} \right)\left( \rho,\eta\right)$ is the tensor expressed in the macroscopic coordinates $\left( I,J,K \right)=\left( X,Y,Z \right)$ with $\chi_{ijk}^{\left( 2 \right)}$ its expression in the frame of the collagen fibre with $\left( i,j,k \right)=\left( x,y,z \right)$. $\left( \boldsymbol{I.i} \right)$ is the scalar projection of $\boldsymbol{I}$ on $\boldsymbol{i}$, which expresses the transformation between the two frames.

Collagen fibres in tissues are formed by a collection of individual fibrils forming larger protofilaments. This global assembly is supposed to be of cylindrical symmetry along a main symmetry axis $z$. The anisotropy of this assembly is expressed similarly as in the tensor of individual collagen fibrils, which symmetry belong to the $C_{6}$ group related to their helical structure. This supposes two independent coefficients $\chi_{zzz}^{\left( 2 \right)}$ (along the main axis $z$ of the cylinder formed by the collagen helix) and $\chi_{zxx}^{\left( 2 \right)}=\chi_{zyy}^{\left( 2 \right)}$ (with $x$ and $y$ the perpendicular directions to $z$). Since the optical coupling is supposed to occur out of resonance, we suppose $\chi_{yzy}^{\left( 2 \right)}=\chi_{yyz}^{\left( 2 \right)}=\chi_{zyy}^{\left( 2 \right)}$ (similarly for coefficients involving $\chi_{zxx}^{\left( 2 \right)}$. Close to resonance, the ratio $\chi_{yzy}^{\left( 2 \right)}/\chi_{yyz}^{\left( 2 \right)}$ becomes an extra unknown parameter of this tensor. Following this model, the $\boldsymbol{I}_{SHG}\left( \alpha\right)$ polarization dependence is only depending on one single parameter, the ratio $\chi_{zzz}^{\left( 2 \right)}/\chi_{zxx}^{\left( 2 \right)}$. This ratio varies typically between 1.2 and 1.6 in collagen type I in tendon tissues [4-6]. Note that in this analysis, the phase of the P-SHG modulation leads to $\rho=\varphi_{2}=\varphi_{4}$

Following this model, and supposing collagen fibres lying in the sample plane $\left( \eta=\pi/2 \right)$, we developed the previous equation into circular functions, to extract the $I_{2}$ parameter that is measured in our experiments. Figure S1B shows the dependence of the $I_{2}$ as a function of $\chi_{zzz}^{\left( 2 \right)}/\chi_{zxx}^{\left( 2 \right)}$ . Ratios varying between 1.2 and 1.6 lead to $I_{2}$ values ranging between 0.15 and 0.4.

(2) *Relation to the distribution* $f\left( \Omega\right).$ The macroscopic tensor $\chi^{(2)}$ is built up from the coherent addition of individual dipoles which molecular tensor components $\beta_{ijk}$ are non-vanishing only in one direction $\boldsymbol{e}$ (typically the red arrows in Fig. S1A), e.g. $\beta_{eee}$. This assumption is valid under non resonant conditions [2, 7]. It is thus possible to define an angular distribution for the elementary dipoles $\boldsymbol{e}$($\theta,\varphi$) which orientation is $(\theta,\varphi)$ in the macroscopic frame. The macroscopic tensor components are thus written:

$\chi_{IJK}^{\left( 2 \right)}=N \left| \beta\right|\int\left( \boldsymbol{I.e} \right)\left( \boldsymbol{J.e} \right)\left( \boldsymbol{K.e} \right)\left( \theta,\varphi\right) f\left( \theta,\varphi\right) sin\theta d\theta d\varphi$

with $\left( \boldsymbol{I.e} \right)$ the scalar projection of $\boldsymbol{I}$ on $\boldsymbol{e}$. $\left| \beta\right|$ is the norm of the $\beta$ tensor.

Supposing that $f\left( \theta,\varphi\right)$ is a cone distribution of average orientation $\left( \rho,\eta\right)$ and angular aperture $\psi$ (Fig. S1A), it is possible to extract the resulting nonlinear tensorial components $\chi_{IJK}^{\left( 2 \right)}$ and to perform a circular decomposition of $\boldsymbol{I}_{SHG}\left( \alpha\right)$. Figure S1B represents the dependence of the anisotropy parameter $I_{2}$as a function of $\psi$, which shows that typical values obtained in collagen type I represent cone apertures $\psi$ of the order of 95°-100°.


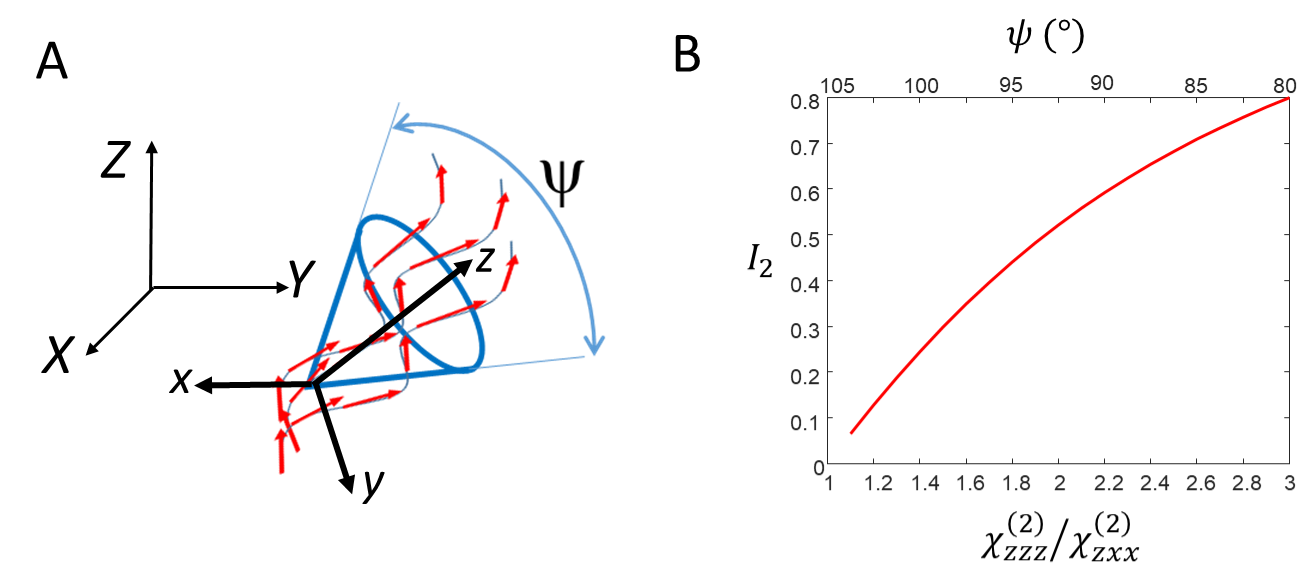


Figure S1. A). schematic representation a thick collagen fibre containing nonlinear dipoles (red arrows) assembled along disordered helices. The macroscopic and fibre axes are represented as well as the resulting angular aperture $\psi$ experienced by the dipoles. B) $I_{2}$coefficient resulting from a P-SHG analysis for different $\chi_{zzz}^{\left( 2 \right)}/\chi_{zxx}^{\left( 2 \right)}$ . ratios and $\psi$ cone apertures.

Note that the cone model evoked above is not necessarily adapted to the physical situation of assemblies of collagen fibrils in tissues. For this reason the data analysis can focus on the anisotropy factor $I_{2}$, which provides a quantification of anisotropy in a medium without the requirement to invoke a given model. In a more general representation, $f\left( \theta,\varphi\right)$ can be decomposed on orders of symmetry which give a more generic quantification of the observed distribution [2].

**Supplementary Materials 2. Influence of the nonlinear induced dipole’s distribution shape and medium birefringence on the SHG polarization dependence and deduced parameters.**

The parameter $I_{4a}$ is in particular useful to quantify any symmetry deviation from the observed angular distribution, since $I_{4a}=0$ for all media of cylindrical symmetry. $I_{4a}$ is for instance non-vanishing anymore in presence of polarization distortions which makes $\varphi_{2}$ and $\varphi_{4}$ become out of phase. This is likely to occur in birefringent media. Collagen and therefore collagen rich tissues such as cartilage are birefringent, with values ranging from $\Delta n=3{.10}^{-3}$ to $7{.10}^{-3}$[8]. Tissue birefringence can complicate the polarization sensitive measurements since the incident polarisation, once focussed inside thick media, does not retain its initial linear direction. This effect increases with depth into the tissue.

Simulations show that the $I_{4a}$ parameter is a relevant reporter of such polarization distortions. Birefringence was modelled in a situation where nonlinear dipoles are organized within a cone distribution of angular aperture ψ, following the approach detailed in [3]. The incident field was no more considered as a linear rotating polarization, but rather as an elliptical polarization, as a result of the birefringent medium passed before reaching the focal plane in the tissue. Birefringence phase shifts values were considered as consistent with a few tens of um depth in collagen, which corresponds to phase shifts δ ranging from 0° to 60°. Using this distorted field in the P-SHG method results in measured ($I_{4a},I_{4s}$) and $I_{2}$ values which are biased. This bias can be visualized in Fig. S2 for different values of δ and all e ψ aperture angles ranging from 1° (very tight organization) to 90° (large aperture). Remarkably, while the $I_{4a}$ parameter remain equal to 0 in the absence of birefringence, it reaches non negligible values in the presence of birefringence (Fig. S2B). We therefore used this signature as a reporter of possible birefringence in the medium.

To monitor whether the measurements were being altered by birefringence in the samples, the parameter $I_{4a}$ was systematically measured (Fig. S2C). Situations that verify $I_{4a}<0.1$ are considered as deprived from birefringence effect, since in this case the effect of birefringence on $I_{2}$ is minimal.


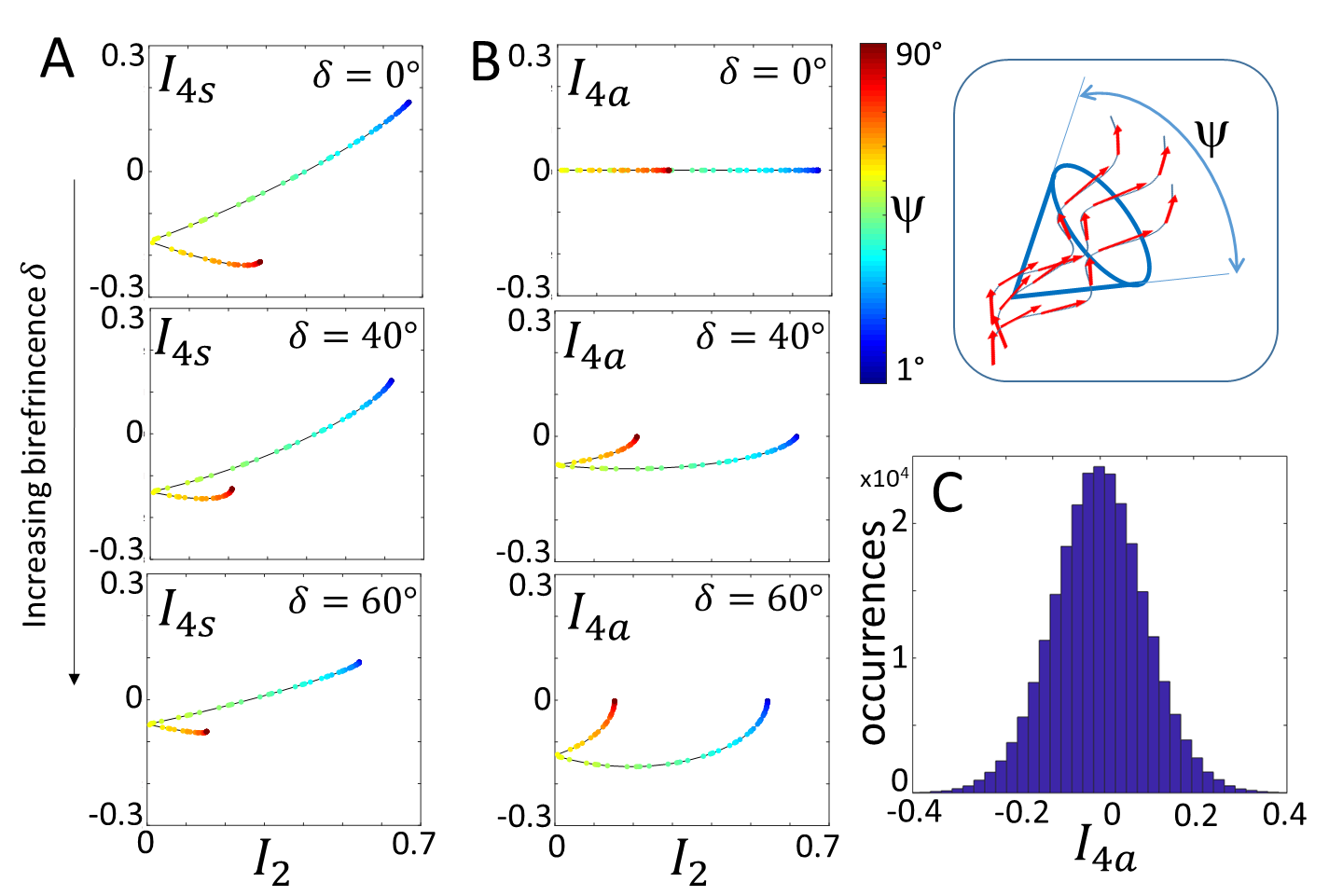


Figure S2. A) Dependence of (I2,I4s) and B) (I2,I4a) values with respect to the angular distribution width (angle ψ) for a cone distribution and for increasing birefringence values corresponding to retardances of 0°, 40°, 60°. These values correspond to typically 0um, 15um, 20um depths considering a birefringence value of Δn = 5. 10^-3^. The inset shows a schematic representation of nonlinear dipoles (red arrows) assembled along disordered helices, similarly as in disorganized collagen filaments in a thick fibre. These dipoles form a cone distribution, of aperture ψ. C) Example of histogram of I4a obtained in a cartilage region at depth below 15 um.

**Supplementary Materials 3. Effect of high numerical aperture excitation and detection on the P-SHG read-out.**

Under a non-paraxial approximation, the expression of the SHG intensity is the integration of the radiated field $\boldsymbol{E}_{SHG}$ over the numerical aperture of the collection objective:

$$\boldsymbol{I}_{SHG}\propto\left| \int_{\zeta=0}^{2\pi} \int_{\sigma=0}^{\sigma_{0}} \boldsymbol{k}\times\boldsymbol{k}\times\boldsymbol{P}_{SHG} \right|^{2}$$

With ($\zeta,\sigma)$ the angular coordinates defining the wave vector $\boldsymbol{k}$ of the propagation direction of $\boldsymbol{P}_{SHG}\propto\chi^{\left( 2 \right)}:\boldsymbol{E}\left( \alpha\right)\boldsymbol{E}\left( \alpha\right)$. $\sigma_{0}$ is the maximum collection angle of the collection objective with $NA=n\sin\sigma_{0}$ its numerical aperture. The expression of the dependence of polarization components mixtures as a function of the numerical aperture used can be adapted from [9].

Since the excitation is also performed at high $NA$, the expression of $\boldsymbol{E}\left( \alpha\right)$ has to account for its axial contribution and $\boldsymbol{E}\left( \alpha\right)=(E_{X}\left( \alpha,\sigma_{0} \right),E_{Y}\left( {\alpha,\sigma}_{0} \right),E_{Z}\left( \sigma_{0} \right))$. With expressions of its components ${(E}_{X},E_{Y},E_{Z})$ that can be found in [10]. Typically, a numerical aperture around 1 such as used in this work leads to an amplitude $E_{Z}$ of about 30% the other in-plane amplitudes.

We suppose that the collection of nonlinear individual dipoles follows a cone distribution of aperture $\psi$, which is potentially out of plane$(\eta=\pi/2).$For simplicity, the macroscopic tensor $\chi^{\left( 2 \right)}$ is first determined in the plane of the sample with $f\left( \Omega\right)$ a cone distribution lying in the sample plane with in-plane orientation $\rho$ relative to $X$. $\boldsymbol{P}_{SHG}$ is then rotated out of plane by an angle $\eta$ relative to $Z$. Fig. S3 represents the bias made on the retrieved coefficient $I_{2}$ when the distribution is tuned out of plane, for a excitation/collection numerical aperture of $NA=1.$ Visibly, the bias starts reaching 20% of the initial value when $\eta<55^{\circ}$ (for small $\psi$ values) and $\eta<45^{\circ}$ (for large $\psi$ values). This means that $I_{2}$ can be reliably determined for tilt angles larger than $\sim50^{\circ}$ with respect to the vertical macroscopic axis.


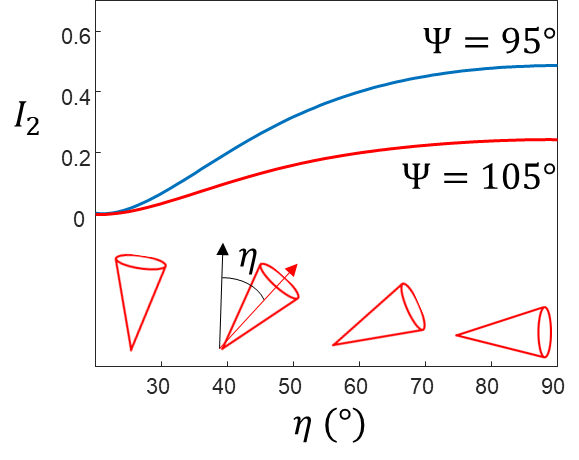


Figure S3. Bias obtained on the ns that $I_{2}$ value for a cone distribution of nonlinear induced dipoles with an angular aperture $\psi$, as a function of the tilt angle $\eta$ with respect to the longitudinal axis $Z.$ The variation of $I_{2}$ is shown for two different $\psi$ values typically measured in collagen type I assemblies in tendons.

**Supplementary Materials 4. P-SHG in collagen type I at the surface of a rat tail tendon.**


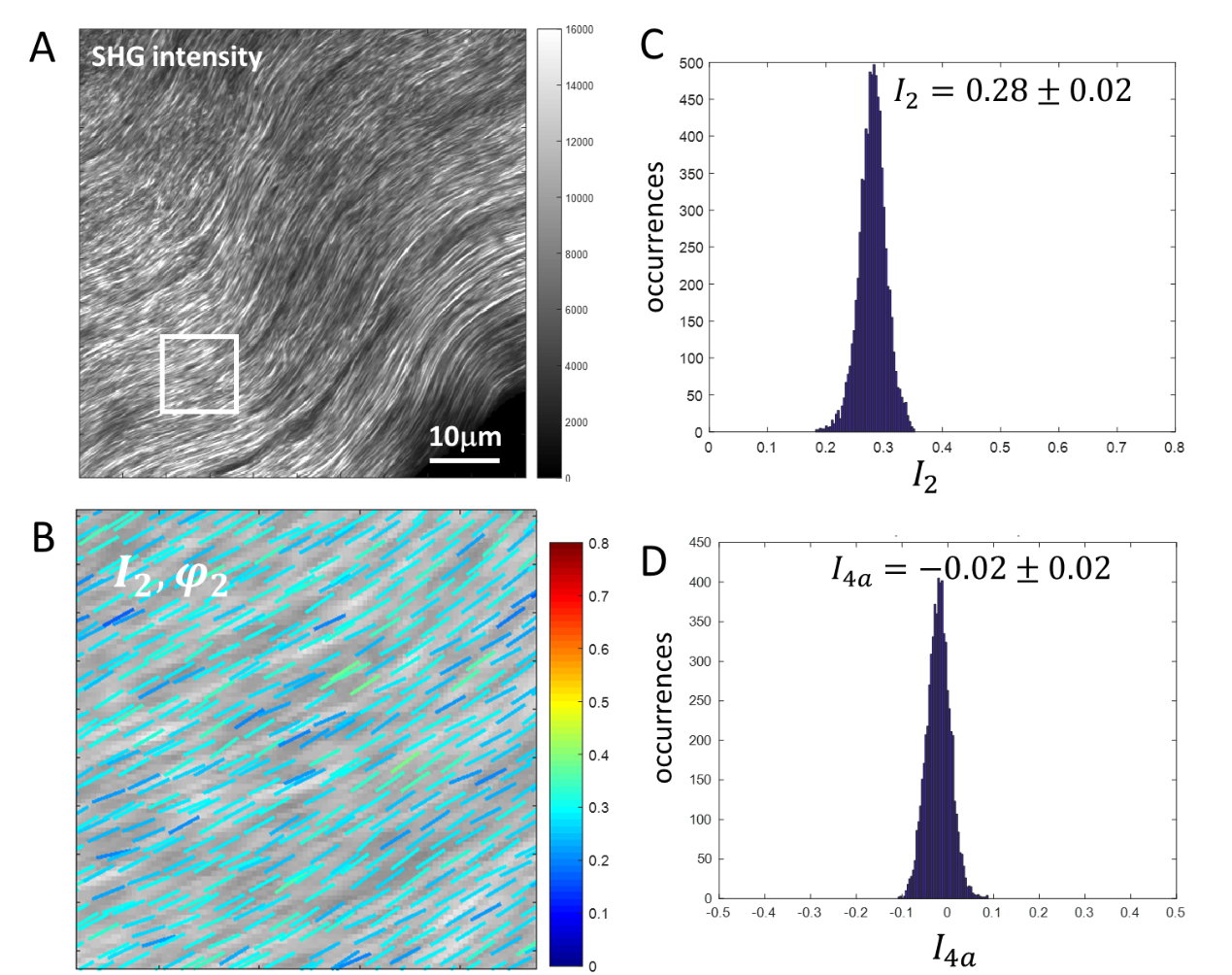


Figure S4. P-SHG performed close to the surface of a rat tail tendon rich in collagen type I. A). Intensity image. B). Map of ${(I}_{2}$,$\varphi_{2}$) represented as sticks coloured with $I_{2}$ and oriented with $\varphi_{2}$, superimposed to the intensity image in grey. C). Statistics of the retrieved $I_{2}$ values for the squared area indicated in B). D) Statistics of the retrieved $I_{4a}$ values for the same area.

**References**

1. Brasselet S. Polarization-resolved nonlinear microscopy: application to structural molecular and biological imaging. Advances in Optics and Photonics 2011; 3: 205.

2. Duboisset J, Aït-Belkacem D, Roche M, Rigneault H, Brasselet S. Generic model of the molecular orientational distribution probed by polarization-resolved second-harmonic generation. Physical Review A 2012; 85: 043829.

3. Ferrand P, Gasecka P, Kress A, Wang X, Bioud F-Z, Duboisset J, et al. Ultimate Use of Two-Photon Fluorescence Microscopy to Map Orientational Behavior of Fluorophores. Biophysical Journal 2014; 106: 2330-2339.

4. Tiaho F, Recher G, Rouede D. Estimation of helical angles of myosin and collagen by second harmonic generation imaging microscopy. Optics Express 2007; 15: 12286-12295.

5. Psilodimitrakopoulos S, Artigas D, Soria G, Amat-Roldan I, Planas AM, Loza-Alvarez P. Quantitative discrimination between endogenous SHG sources in mammalian tissue, based on their polarization response. Optics Express 2009; 17: 10168-10176.

6. Gusachenko I, Tran V, Houssen YG, Allain JM, Schanne-Klein MC. Polarization-Resolved Second-Harmonic Generation in Tendon upon Mechanical Stretching. Biophysical Journal 2012; 102: 2220-2229.

7. Davis RP, Moad AJ, Goeken GS, Wampler RD, Simpson GJ. Selection rules and symmetry relations for four-wave mixing measurements of uniaxial assemblies. The Journal of Physical Chemistry B 2008; 112: 5834-5848.

8. van Turnhout M, Kranenbarg S, van Leeuwen J. Modeling optical behavior of birefringent biological tissues for evaluation of quantitative polarized light microscopy. Journal of Biomedical Optics 2009; 14: 054018.

9. Axelrod D. Fluorescence excitation and imaging of single molecules near dielectric‐coated and bare surfaces: a theoretical study. Journal of microscopy 2012; 247: 147-160.

10. Chon JW, Gan X, Gu M. Splitting of the focal spot of a high numerical-aperture objective in free space. Applied physics letters 2002; 81: 1576-1578.
