# Supplementary material for: Collagen reorganization in cartilage under strain probed by polarization sensitive second harmonic generation microscopy
Source: J R Soc Interface. 2019 Jan 16;16(150):20180611. doi: 10.1098/rsif.2018.0611 (PMC6364654; doi:10.1098/rsif.2018.0611)
Supplement: supplementary materials 5 [file rsif20180611supp2.pptx]

## Slide 1
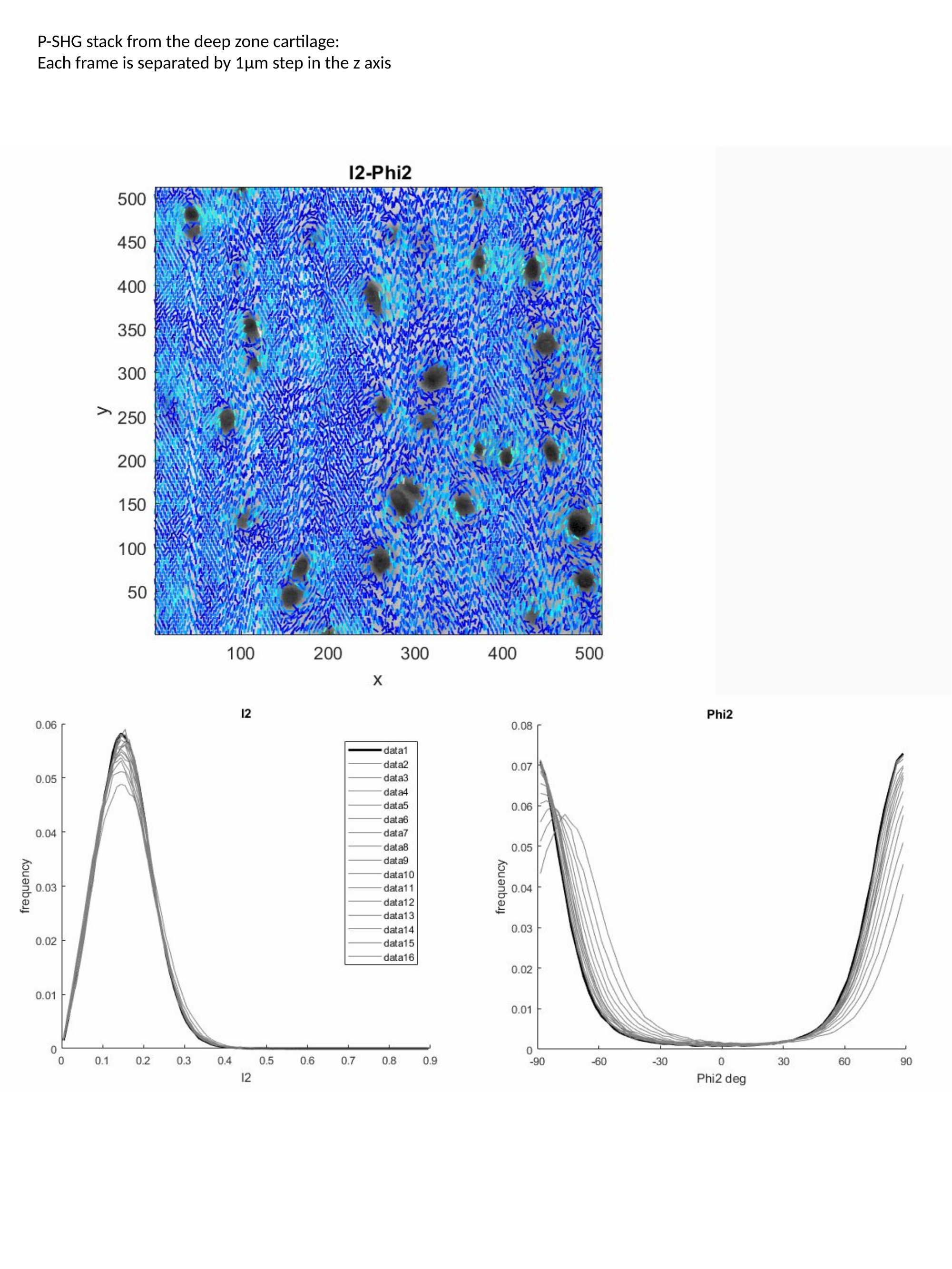

P-SHG stack from the deep zone cartilage:
Each frame is separated by 1μm step in the z axis

## Slide 2
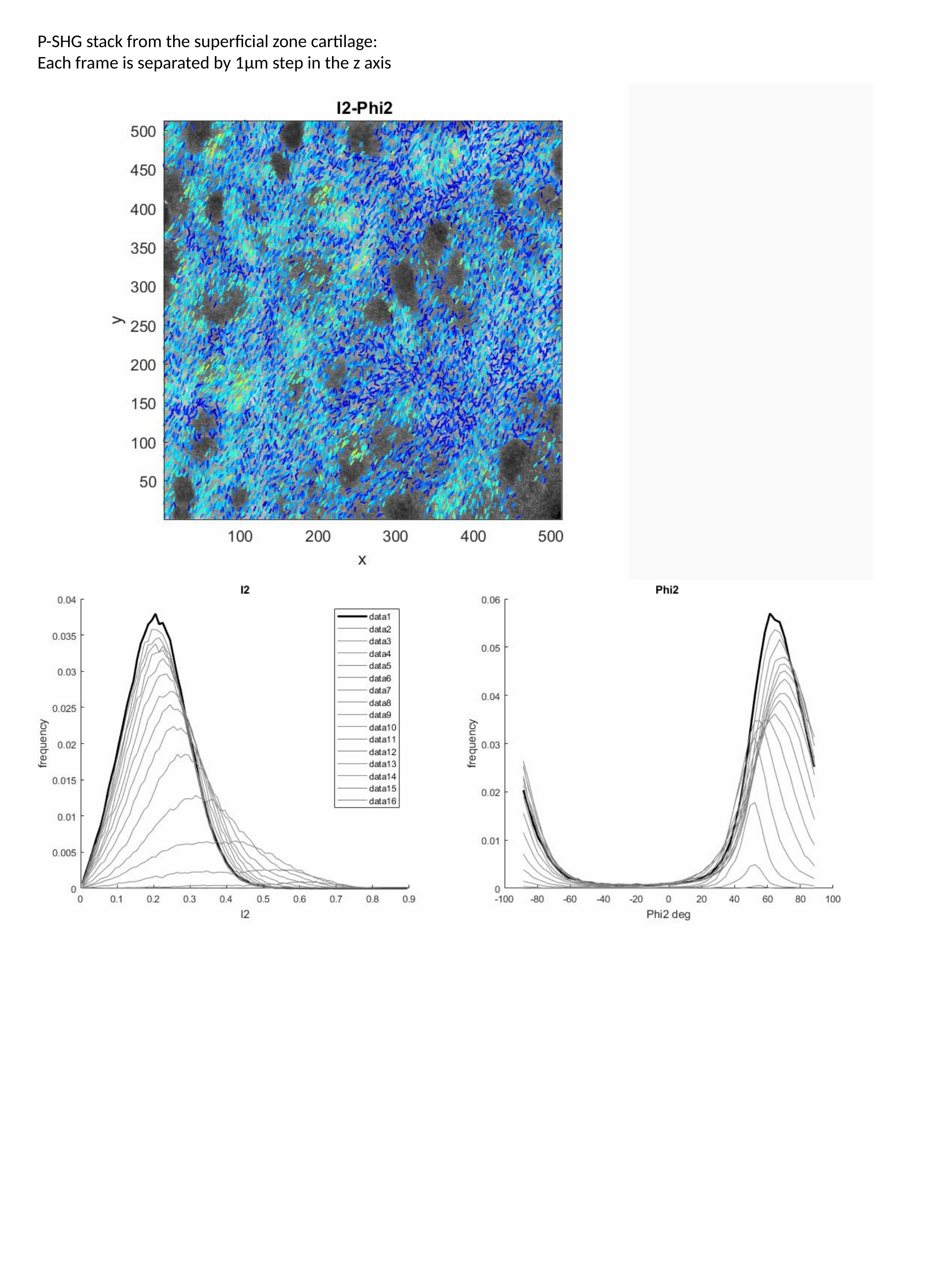

P-SHG stack from the superficial zone cartilage:
Each frame is separated by 1μm step in the z axis
